# The PaleoJump database for abrupt transitions in past climates

**DOI:** 10.1038/s41598-023-30592-1

**Published:** 2023-03-18

**Authors:** Witold Bagniewski, Denis-Didier Rousseau, Michael Ghil

**Affiliations:** 1 Department of Geosciences and Laboratoire de Météorologie Dynamique (CNRS and IPSL), École Normale Supérieure, PSL University, Paris, France; 2 Geosciences Montpellier, CNRS, University of Montpellier, Montpellier, France; 3 Institute of Physics - CSE, Division of Geochronology and Environmental Isotopes, Silesian University of Technology, Gliwice, Poland; 4 Lamont-Doherty Earth Observatory, Columbia University, New York, USA; 5 Department of Atmospheric and Oceanic Sciences, University of California at Los Angeles, Los Angeles, USA

**Keywords:** Palaeoclimate, Nonlinear phenomena, Scientific data

## Abstract

Tipping points (TPs) in Earth’s climate system have been the subject of increasing interest and concern in recent years, given the risk that anthropogenic forcing could cause abrupt, potentially irreversible, climate transitions. Paleoclimate records are essential for identifying past TPs and for gaining a thorough understanding of the underlying nonlinearities and bifurcation mechanisms. However, the quality, resolution, and reliability of these records can vary, making it important to carefully select the ones that provide the most accurate representation of past climates. Moreover, as paleoclimate time series vary in their origin, time spans, and periodicities, an objective, automated methodology is crucial for identifying and comparing TPs. To address these challenges, we introduce the open-source PaleoJump database, which contains a collection of carefully selected, high-resolution records originating in ice cores, marine sediments, speleothems, terrestrial records, and lake sediments. These records describe climate variability on centennial, millennial and longer time scales and cover all the continents and ocean basins. We provide an overview of their spatial distribution and discuss the gaps in coverage. Our statistical methodology includes an augmented Kolmogorov–Smirnov test and Recurrence Quantification Analysis; it is applied here, for illustration purposes, to selected records in which abrupt transitions are automatically detected and the presence of potential tipping elements is investigated. These transitions are shown in the PaleoJump database along with other essential information about the records, including location, temporal scale and resolution, as well as temporal plots. This open-source database represents, therefore, a valuable resource for researchers investigating TPs in past climates.

Since the discovery of Dansgaard-Oeschger (DO) events in Greenland ice cores^1–3^, climate research has aimed to identify other examples of centennial-to-millennial climate variability, including in marine and terrestrial paleoclimate records^4,5^, and to gain insight into the mechanisms behind these changes. Many such records exhibit abrupt transitions, raising the question of whether similarly drastic changes may occur in the near future, as anthropogenic global warming pushes the climate system away from the relatively stable state that has persisted throughout the Holocene. Many of Earth’s subsystems exhibit intrinsic variability and respond nonlinearly to various natural and anthropogenic forcings^6,7^. Hence, any of these subsystems could experience a sudden shift into a new state once certain key thresholds, known as tipping points (TPs), are crossed^8–10^.

Identifying potential TPs in the climate system requires theoretical and modeling work, including comparison with observations. Proxy records of past climate and environmental changes play a crucial role, by enabling the reconstruction of Earth’s climatic history. These records, particularly those that show abrupt transitions, may provide valuable insights into the past behavior of Earth’s systems and possible TPs. By using this information, we can gain a better understanding of the possible trajectories of future climate change and their environmental impacts.

With tens of thousands of paleoclimate datasets available, finding and selecting the most relevant records for studying past climate can be overwhelming. These proxy datasets originate from different geologic structures, contain different variables, and span a wide range of age intervals with different resolutions. In addition, due to the varying resolution and dating methods used for these records, it is important to assess their reliability. There have been efforts in recent years to build compilations of high-quality paleoclimate data, such as the ACER database of pollen and charcoal records from the last glacial period^11^, the SISAL database of speleothem isotope records^12^, the PalMod compilation of marine sediment data covering the last glacial-interglacial cycle^13^, the World Atlas of late Quaternary Foraminiferal Oxygen and Carbon Isotope Ratios^14^, and the PhanSST database of Phanerozoic sea surface temperature proxy data^15^. These compilations, however, are typically limited to a specific type of proxy or time interval.

To fully understand TPs, which can affect various components of the earth system^6,9,16^ and have occurred at different times in the past, it is essential to examine a diverse range of paleoclimate data. This includes marine, terrestrial, and ice records from different time intervals during which abrupt climate change events have occurred, such as the Paleocene-Eocene Thermal Maximum (PETM), DO events, and the Younger Dryas. Furthermore, as tipping elements—the subsystems that may be subject to tipping—are interconnected, a potential for domino effects exists^17^. To identify and describe such effects in past records, one needs coverage from different types of archives with a comprehensive geospatial distribution.

The purpose of this paper is to address these challenges by presenting the open-source PaleoJump database^18^, https://paleojump.github.io, which compiles globally sourced high-resolution paleoclimate records that contain abrupt transitions. The records originate in ice cores, marine sediments, speleothems, terrestrial deposits, and lake sediments, and can provide valuable information for modeling and predicting critical TPs in current and future climate evolution. The database is designed as a website, allowing easy access and navigation. It includes a map of the paleoclimate records, as well as tables that list supplementary information for each record, along with dates of the detected transitions.

In addition to providing a description of the PaleoJump database, we demonstrate its potential use by conducting a TP analysis of seven records selected from the database. Since paleoclimate records vary in their origin, time spans, and periodicities, an objective, automated methodology is key for identifying and comparing TPs. Here, we apply a recently developed method to detect abrupt transitions based on an augmented Kolmogorov–Smirnov (KS) test^19^ to the selected records. The KS test results are then compared with those of recurrence quantification analysis (RQA)^20,21^ to further assess the validity of the findings.

The next section describes the database itself, followed by a section summarizing the KS and RQA methods. Next, a section illustrates the application of these two methods to the seven selected records, including comparisons between the results for distinct records. The following section compares the results of the two methods and provides an interpretation of our findings. Conclusions of the work follow in the last section.

## Database sources

The PaleoJump database currently includes records from 131 sites, grouped by their geological type: 49 marine-sediment cores, 32 speleothems, 18 lake sediment cores, 20 terrestrial records, and 12 ice cores. The main sources for this database are the PANGAEA and NCEI/NOAA open-access data repositories, while some records are, unfortunately, available only on request from the authors of the original studies; in the latter case, links to the corresponding articles are provided. The paleorecords have been selected for their ability to represent different aspects of past climate and environmental variability. In addition to containing abrupt transitions, these records are characterized by high temporal resolution, multi-millennial time scales, and a comprehensive spatial coverage. This selection simplifies the search for records that are most helpful in the investigation of critical transitions and of the behavior of tipping elements.

Resolution is a crucial factor in the selection of records. As a metric, we use ‘maximum resolution’, which we define as the temporal resolution of the 10 ky interval with the smallest average time step. We took this approach to ensure that records with nonuniform resolution or with gaps in the data are not overlooked. To make the selection more conservative, we only considered resolution for time intervals older than 14 ka BP because more recent intervals often have a significantly higher resolution. The selection criteria varied depending on the source type and time interval. For example, for records that only cover the Last Glacial Cycle, we select those with a maximum resolution of 200 years or better, while older records in our database have a resolution no worse than 400 years. However, exceptions were made for records from undersampled regions, such as the Guliya ice core^22^ and several lake sediment records, and for exceptionally long and complete records, like the CENOGRID^23^ and Chinese Loess Plateau^24^ stacks.

While many of the paleosites listed in PaleoJump include multiple proxy types, we have focused on proxies that can be directly compared with climate models: oxygen isotopes reflecting changes in past temperatures, sea level, and precipitation; carbon isotopes containing information on past vegetation and the carbon cycle; aeolian deposits that include signatures of past precipitation, mineral aerosols, and atmospheric transport patterns; as well as other proxy-based estimates of past temperatures. We have mainly focused on the Last Glacial Cycle, due to the well-established evidence of past abrupt transitions—such as DO and Heinrich events—with most records also covering Holocene deglaciation. Other records extend further back in time, including DO-like events during earlier glacial cycles of the Quaternary, and earlier climatic events of the Cenozoic era, such as the Eocene–Oligocene Transition at 34 Ma or the PETM at 56 Ma. While PaleoJump provides global coverage with records from all continents and ocean basins, its spatial coverage is biased towards the North Atlantic region due to greater availability and to the strong impact of the DO events.

The well-known uncertainties in paleoclimate records need to be considered in building such a database. A key source of uncertainty is in their chronologies, as dating methods such as radiocarbon dating and layer counting have limitations and potential sources of error^25,26^. When independent dating is unavailable, “wiggle matching” is often performed to match records with other, well-dated records, but this method’s use can be complicated by differences in local climate variability and time lags in climate signal propagation^27,28^. Additionally, recorded values of climate proxies may be affected by extra-climatic processes—physical, biological, and chemical—or by measurement errors. Interpretation of proxy records can also be challenging because a given proxy variable may be influenced by multiple climatic processes^29–31^, making it difficult to determine the specific climate conditions it represents.

In many of the records in PaleoJump, the chronology used for the original study has since been updated in more recent ones. When this is the case, we use the most recent age model’s chronology. However, when the newer chronology does not cover the complete record, which is often the case, we use the older chronology. Furthermore, we did not attempt to harmonize and synchronize the age models among the records. These choices ensure that the most complete versions of the records are included in the database, while also allowing users to independently reproduce our analyses without having to reevaluate how harmonization was performed.

**Figure 1:**
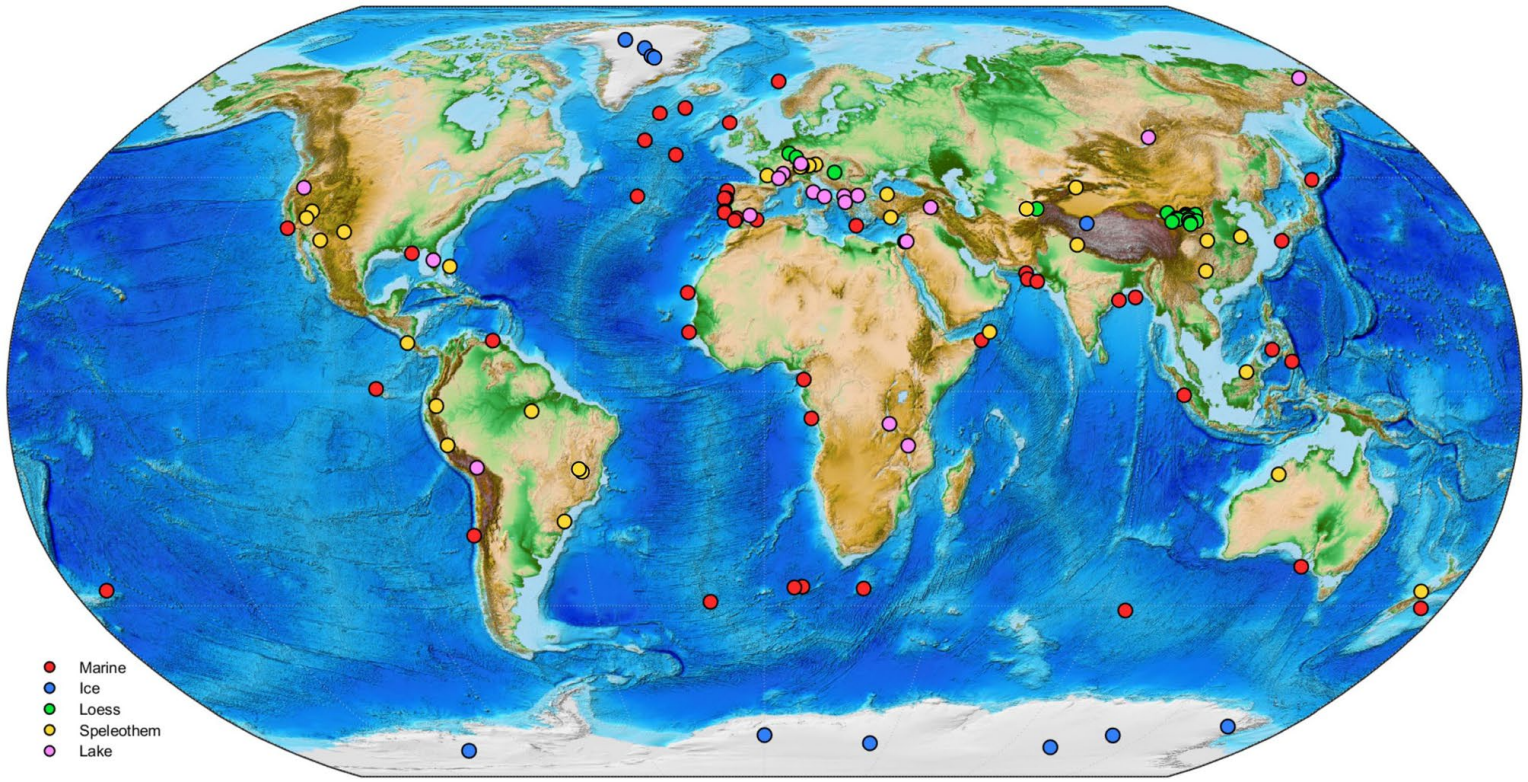
Map of the records included in the PaleoJump database and listed in the Supplementary Information. The five record sources—marine sediments, ice cores, terrestrial deposits, speleothems, and lake sediments—are identified in the legend by color. The land topography and ocean bathymetry are based on the ETOPO1 Global Relief Model data^175^.

Five tables show the information for each record in the PaleoJump database and are included in the Supplementary Information^4,5,11,22–24,32–174^ (Fig. 1).

## Applying the KS test and RQA to TP identification

Given the diversity of the proxy records in the PaleoJump database, an objective, automated methodology is crucial for identifying and comparing TPs. Bagniewski et al.^19^ formulated an augmented KS test, which they used to robustly detect and identify abrupt transitions during the last glacial cycle. These results were compared with results obtained using RQA, showing that the two methods are complementary, with KS test being more useful at detecting individual jumps and determining their exact dates, while RQA can help establish important transitions in a record’s characteristic time scale. Here, we apply these two methods to a selection of records from the PaleoJump database and demonstrate the ability of the augmented KS test to accurately identify transitions in various types of paleoclimate records.

### Kolmogorov–Smirnov (KS) test

The augmented KS methodology^19^ is based on the nonparametric KS test^176^. A two-sample KS test is applied to two neighboring samples drawn from a proxy time series within a sliding window of length *w*. The commonality of the two samples is quantified by the the KS statistic $D_\mathrm{{KS}}$^176,177^. A “jump” in the time series is identified at any point in time at which $D_\mathrm{{KS}}$ is greater than a cut-off threshold $D_\text{c}$. As the KS test can give very different results depending on the window length *w* being used, $D_\mathrm{{KS}}$ is calculated for different *w*’s, varying between $w_\mathrm{{min}}$ and $w_\mathrm{{max}}$. The values of the latter two parameters bracket the desired time scale at which a given paleorecord is to be investigated. Furthermore, smaller jumps in the time series may be the result of an error in the observed data or small-scale variability that occurs over time intervals shorter than the sampling resolution of the proxy record and they should be discarded. Thus, for a transition to be considered significant, the change in magnitude between the two samples (*i*, *j*) should exceed a threshold $\sigma _\text{c}$ in their standard deviations $(\sigma _i, \sigma _j)$. Finally, as the KS test requires a large enough sample size to be significant, its results are rejected if either of the two samples has a size *n* smaller than $n_\text{c}$.

At a time step at which all three conditions based on the parameters $D_\text{c}$, $\sigma _\text{c}$, and $n_\text{c}$ are satisfied, an abrupt transition is identified. As the dates of such transitions often occur in clusters, the precise date for a transition within such a cluster is determined by the maximum $D_\mathrm{{KS}}$ value found within the corresponding time interval. When the maximum $D_\mathrm{{KS}}$ over a given interval is shared by several time steps, the one corresponding to the maximum change in absolute magnitude is used; moreover, if there are several jumps of equal amplitude, then the one with the earliest date is used.

As the same transition may be found at slightly different dates depending on the window length that is used, we first identify the transitions detected with the longest window, which, given the larger sample size it accommodates, is the most statistically significant one. These transitions are then supplemented by those detected for the next-longest window and eventually for all other window lengths. For window $w_i$, we discard transitions identified at time *t* if the interval $\{t - w_i \le t \le t + w_i\}$ contains transitions that were previously identified with a greater window length. Finally, to identify transitions between dominant climate modes, such as the Stadial-Interstadial (GS–GI) boundaries, we use a running window to extract the upper and lower values from the time series and locate the transitions that mark a shift from one mode to the other.

The parameters $D_\text{c}$ and $\sigma _\text{c}$ are initially optimized following receiver operating characteristic analysis^178,179^, and abrupt transitions so identified for the NGRIP ice core δ^18^O record are further compared with the change points identified using visual inspection by Rasmussen et al*.*^131^.

### Recurrence Quantification Analysis (RQA)

The KS test results are next compared with RQA results^20,21,180^. Here, the Recurrence Plot (RP) for a time series $\{x_k: k = 1, \ldots , K\}$ is given by a square pattern in which both axes represent time. A dot is entered into a position (*i*, *j*) of the matrix $\textbf{R}$ when $|x_i - x_j| < \varepsilon$, with $\varepsilon$ being the recurrence threshold. Thus, the RP appears as a square matrix $\textbf{R}$ of dots. For details on how $\varepsilon$ is determined, see Marwan et al*.*^21^ and Bagniewski et al*.*^19^.

Eckmann et al.^20^ showed that purely visual RP typologies provide useful information about a time series. However, RQA allows for a more objective way of inferring recurrence^21,180^, by quantifying selected recurrence characteristics. One of the simplest RQA criteria is the recurrence rate (RR), namely the density of dots within the recurrence plot: RR describes the probability of states of the system recurring within a particular time interval. By evaluating RQA measures such as RR in a sliding window, it is possible to identify changes in the time series. Low RR values correspond to an unstable behavior of the system, and hence abrupt transitions in a time series may be identified by local RR minima.

An important advantage of the recurrence method is that it does apply to dynamical systems that are not autonomous, i.e., that may be subject to time-dependent forcing^20^. The latter is certainly the case for the climate system in general^181–184^ and, in particular, on the time scales of 10-100 kyr and longer, which are affected strongly by orbital forcing^185^.

For a more comprehensive description of both the augmented KS test and RQA, see Bagniewski et al.^19^.

## Examples of usage

Here we show the results of the augmented KS test methodology^19^, as applied to records of different timescales, resolutions, and periodicities. Plots of the detected transitions, along with files listing their dates are included in the PaleoJump database; the same is available for other records as well.

### Methodology

We apply the augmented KS test to six records, given in Table 1, from each of the proxy types listed in Supplementary Tables 1–5. In addition to these six records, we include the results obtained for the NGRIP ice core, which have been published in Bagniewski et al.^19^, and compare the latter with the three records of the last glacial cycle in the table, to wit MD03-2621, Paraiso Cave, and ODP893A.

**Table 1:** Records analyzed using the augmented KS test^19^, ordered by geological type and temporal scale; the nature of the records is indicated in the last column by the abbreviations ben = benthic, pla = planktonic, and TIC = Total Inorganic Carbon.

Type	Site name	Location	Depth/elevation	Age	Res.	Proxy
Marine	ODP893A^87,88^	34.28, -120.03	576 m	65–0 ka	41 y	pla $\delta ^{18}$O
Marine	MD03-2621^64^	10.678, -64.972	847 m	109–6 ka	0.1 y	reflectance
Marine	U1308^95^	49.878, -24.238	3871 m	3143–0 ka	118 y	ben $\delta ^{18}$O
Marine	CENOGRID^23^	N/A	N/A	67.1–0 Ma	2000 y	ben $\delta ^{18}$O
Terrestrial	Paraiso (PAR07)^165^	-4.067, -55.45	60 m	45–18 ka	21 y	$\delta ^{18}$O
Terrestrial	Lake Ohrid^137^	41.049, 20.715	693 m	1.36–0 Ma	208 y	TIC
Ice	NGRIP^131^	75.1, -42.32	2925 m	122–0 ka	20 y	$\delta ^{18}$O

The KS test parameters vary depending on a record’s time resolution and on the length of its age interval. For the records covering the last glacial cycle (MD03-2621, Paraiso Cave, and ODP893A), we use the same parameter values as used for the NGRIP record in Bagniewski et al.^19^, i.e., $D_\text{c} = 0.77$, $\sigma _\text{c} = 1.9$, $n_\text{c} = 3$, $w_\mathrm{{min}} = 0.1$ kyr, and $w_\mathrm{{max}} = 2.5$ kyr. For the records spanning longer time intervals with a lower temporal resolution, we use longer window lengths $w_\mathrm{{min}}$ and $w_\mathrm{{max}}$, thus shifting the focus of our analysis to longer time scales. For the U1308 benthic $\delta ^{18}$O and Lake Ohrid TIC records, we use a *w*-range of 2 kyr to 20 kyr. This allows us to focus on the glacial-cycle variability, as the record’s resolution is too low to properly identify DO events, particularly for data older than 1.5 Ma BP, when the U1308 record’s resolution is lower than for more recent data. For the CENOGRID record, we perform two separate analyses, one with a *w*-range of 1 Myr to 4 Myr to determine the Quaternary’s major climatic shifts, and one with a *w*-range of 0.02 Myr to 2.5 Myr, which covers the orbital time scale. Note that, in paleoclimate studies, one distinguishes between units of absolute time, such as kyr or Myr, and units of age, such as ka BP or Ma BP, where ‘BP’ stands for “before present.”

### Results for individual records

**Figure 2:**
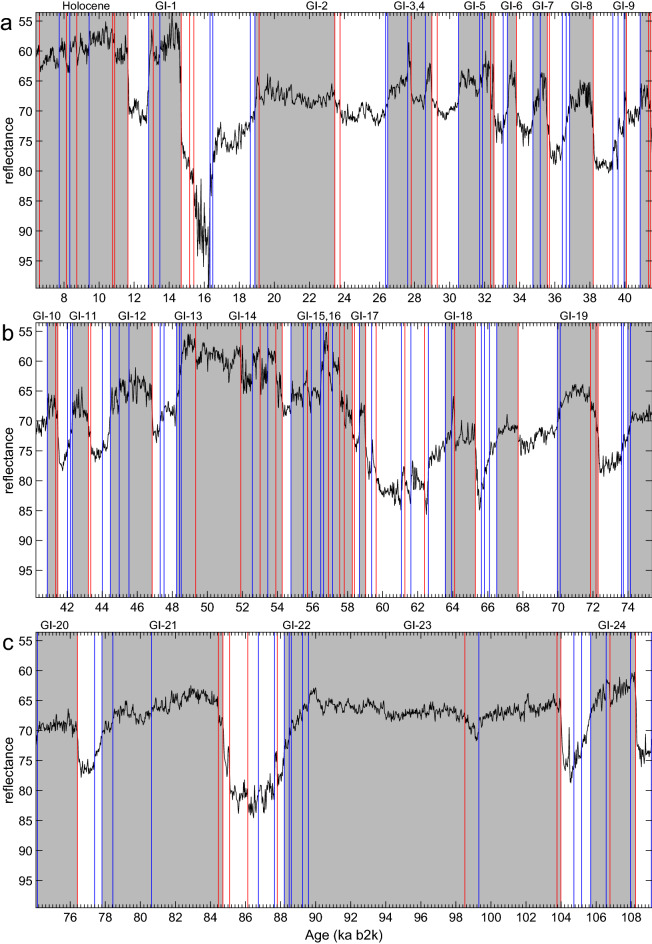
MD03-2621 marine sediment core: (**a**) 6.4–41.6 ka b2k; (**b**) 40.2–75.4 ka b2k; and (**c**) 74–109.2 ka b2k. The black solid line is the core reflectance^64^, with the vertical axis reversed. Vertical lines represent transitions detected by the KS test of Bagniewski et al.^19^, with colors indicating the direction of the jumps. In this figure, red lines represent a warming event, while blue lines represent a cooling event. In other figures, the meaning of the colors may vary. Grey bars denote warm episodes. Note that all time axes in this paper follow the geological custom of pointing into the past.

We chose the MD03-2621 reflectance record from the Cariaco Basin in the Caribbean for having a very high resolution and for its importance in studying the effect of DO events on the migrations of the intertropical convergence zone (ITCZ). The record is shown in Fig. 2 and it has been used previously to assess teleconnections between the North Atlantic basin and the Arabian Sea^64^. When the ITCZ migrates southward during stadials, northeasterly Trade winds lead to upwelling of cool, nutrient-rich waters in the Caribbean; as the ITCZ migrates northward during interstadials, heavy convective rainfall leads to increased runoff from South America’s north coast, delivering detrital material to the Cariaco Basin^64,186^. As a result, the color reflectance in the core alternates between light-colored sediments, rich in foraminiferal carbonate and silica, and darker sediments abundant in detrital organic carbon. These changes in the marine sediments are proxies for the prevailing atmospheric circulation regime, and the meridional position of the ITCZ in the region, which are both linked to the glacial-interglacial variability.

For the KS analysis, a 20-year moving average of the MD03-2621 record is calculated in order to align its resolution with that of the NGRIP record. Our analysis does identify the “classical” DO events, as seen in the NGRIP record^19,131^. There is, however, no direct relationship in the identified longer-scale warm (grey bars) and cool intervals: For instance, some events appear to be merged in Fig. 2, e.g., GI-23 and 22, GI-16 and 15, GI-14 and 13, GI-10 and 9, and GI-4 and 3, while some events detected here, between 68 and 66 ka BP, are not identified in NGRIP, and GI-2 is much longer than in NGRIP.

**Figure 3:**
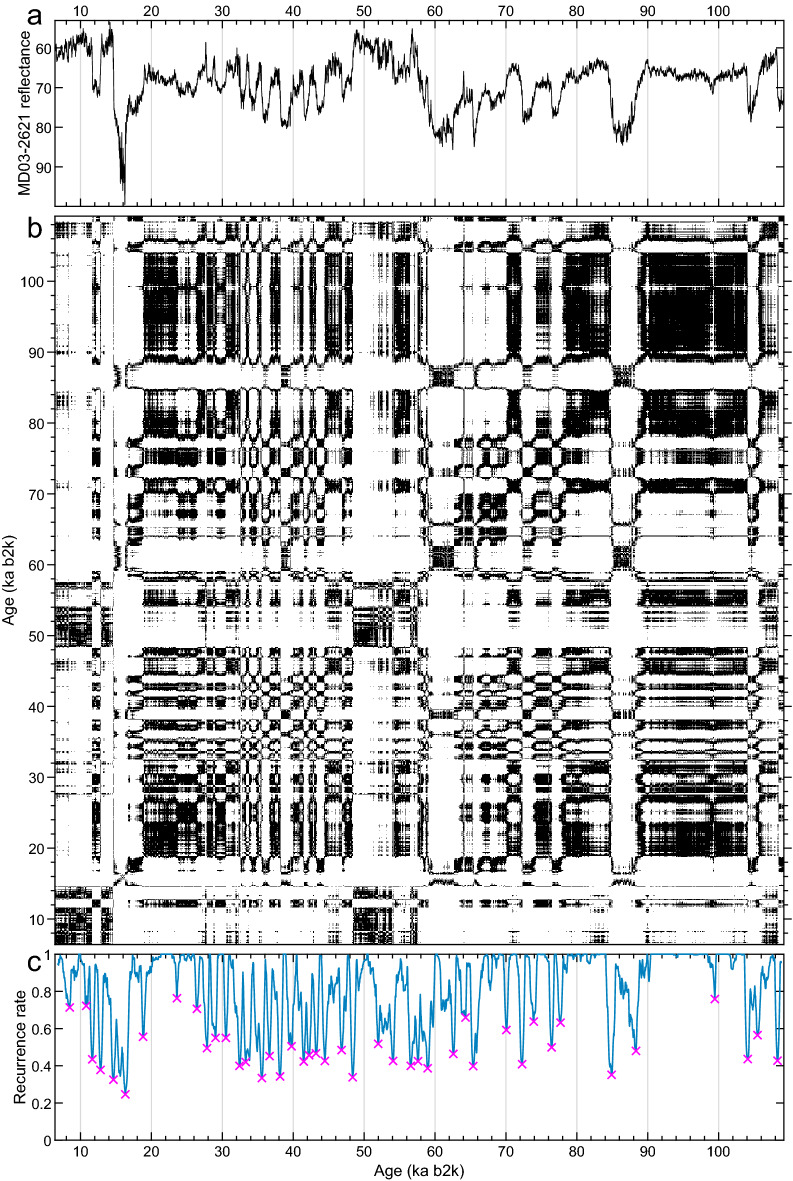
Recurrence Quantification Analysis (RQA)^21,180^ for the MD03-2621 reflectance record^64^. (**a**) Time series with vertical axis as in Fig. 2; (**b**) recurrence plot (RP); and (**c**) recurrence rate. Magenta crosses in (**c**) represent the transitions identified by the RQA.

The RQA analysis^21,180^, shown in Fig. 3, does identify the major transitions in the MD03-2621 reflectance record, including the relative significance of each. It does not, however, resolve smaller transitions that occur at the centennial time scale. Please see Bagniewski et al.^19^ for the explanation of the recurrence rate used to identify the transitions in the figure’s panel (c).

**Figure 4:**
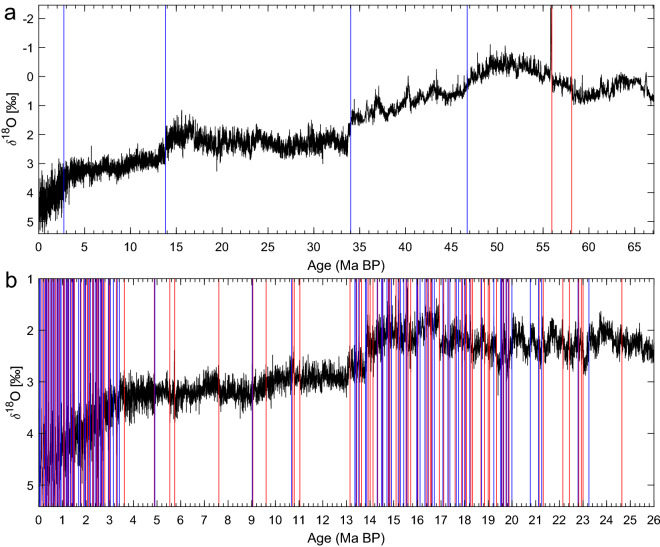
CENOGRID stack of benthic $\delta ^{18}$O^23^. Vertical lines represent transitions detected by the KS test^19^, with colors indicating the direction of the jumps. Grey bars represent warm episodes. (**a**) Transitions detected for the entire record using a window *w* range of 1–4 Myr, and (**b**) transitions detected for the interval 26–0 Ma BP using a *w* range of 0.02–2.5 Myr; see also the earlier subsection on “Methodology.” The vertical axes are reversed.

The CENOGRID stack of benthic $\delta ^{18}$O^23^ shown here in Fig. 4 is a highly resolved 67 Myr composite from 14 marine records. Westerhold et al.^23^ distinguished four climate states—Hothouse, Warmhouse, Coolhouse, and Icehouse—in this record, largely following changes in the polar ice volume. The composite in the figure^23^ reconstructs in detail Earth’s climate during the Cenozoic era, at a higher time resolution than the earlier compilation of Zachos and colleagues^187^.

Our KS analysis in Fig. 4a uses a window width 1$\le w \le$4 Myr and it identifies four major transitions towards higher $\delta ^{18}$O values and two towards lower ones. The oldest transition, at 58 Ma BP, is characterized by a shift from the moderately warm climate prevailing at the beginning of the Cenozoic to the hot conditions marked by the Early Eocene Climate Optimum between 54 Ma and 49-48 Ma BP. The second transition corresponds to the short but intense warm event known as the PETM, the Cenozoic’s hottest one. The third transition marks the end of this hot interval and the return to the milder and relatively stable conditions that prevailed between 67 Ma and 58 Ma BP.

The fourth transition, at 34 Ma, is the Eocene–Oligocene Transition, the sharp boundary between the warm and the hot climatic conditions in the earlier Cenozoic and the Coolhouse and then Icehouse conditions prevailing later on^188^. As this transition marks a major shift in the Earth’s climate dynamics, it is a candidate for a major TP in Earth’s climate history^189^. The fifth transition, at 14 Ma BP, ends a rather stable climate interval between 34 Ma and 14 Ma, characterized by the build up of the East Antarctic ice sheet^190,191^. This transition also marks the start of an increasing trend in benthic $\delta ^{18}$O values^192,193^. The final transition marks the start of the Icehouse world and it is characterized by the emergence and development of the Northern Hemisphere ice sheets.

Using a reduced window length on the last 26 Myr of the CENOGRID benthic record, many more abrupt transitions are detected in Fig. 4b. In particular, higher variability in the $\delta ^{18}$O signal and much more frequent transitions are found during two intervals, namely 71 transitions over the last 3.5 Myr and 77 transitions between 20 Ma BP and 13 Ma BP. In contrast, the intervals 67–20 Ma BP and 13–3.5 Ma BP are characterized by a lower frequency of detected transitions, with 112 and 13 transitions, respectively. The former one of the two intervals with many jumps includes the Quaternary Period, which started 2.6 Myr ago and is well known for its high climate variability, due to the presence of large ice masses in the system^6,194^.

The interval 20–13 Ma BP, on the other hand, coincides with the exclusive use of the U1337 and U1338 records in constructing the CENOGRID stack, both of which are located in the eastern tropical Pacific. Higher sedimentation rates in these two cores might contribute to the higher variability $\delta ^{18}$O observed in the stack record over this interval. For transitions detected with the shorter window for the entire CENOGRID stack, please see Fig. S1 in the Supplementary Information.

**Figure 5:**
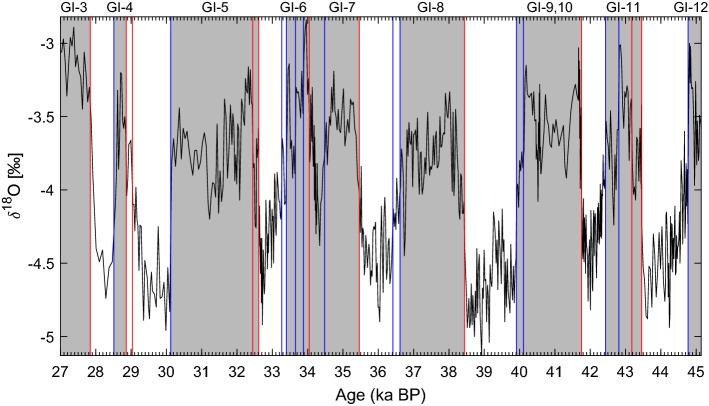
Paraiso Cave (PAR07) $\delta ^{18}$O^165^. Vertical lines represent transitions detected by the KS algorithm^19^, with colors indicating the direction of the jumps. Grey bars represent dry interglacial conditions.

The $\delta ^{18}$O record from the Paraiso Cave in the Amazon rainforest reveals a relationship between shifts in global temperature and changes in rainfall patterns in the region; see Fig. 5. The record shows that the Amazon basin experienced drier conditions during the last glacial period, most probably due to the lower temperatures. The record exhibits reduced precipitation during DO interstadials and increased precipitation during stadials, a pattern that negatively correlates with Chinese speleothem records^55^, suggesting a phase opposition in rainfall between the two regions. The abrupt shifts in precipitation during DO events suggest that the Amazonian climate subsystem may exhibit bistability, making it a potential tipping element as changes in precipitation may lead to forest dieback^195^.

### Results for core intercomparisons

**Figure 6:**
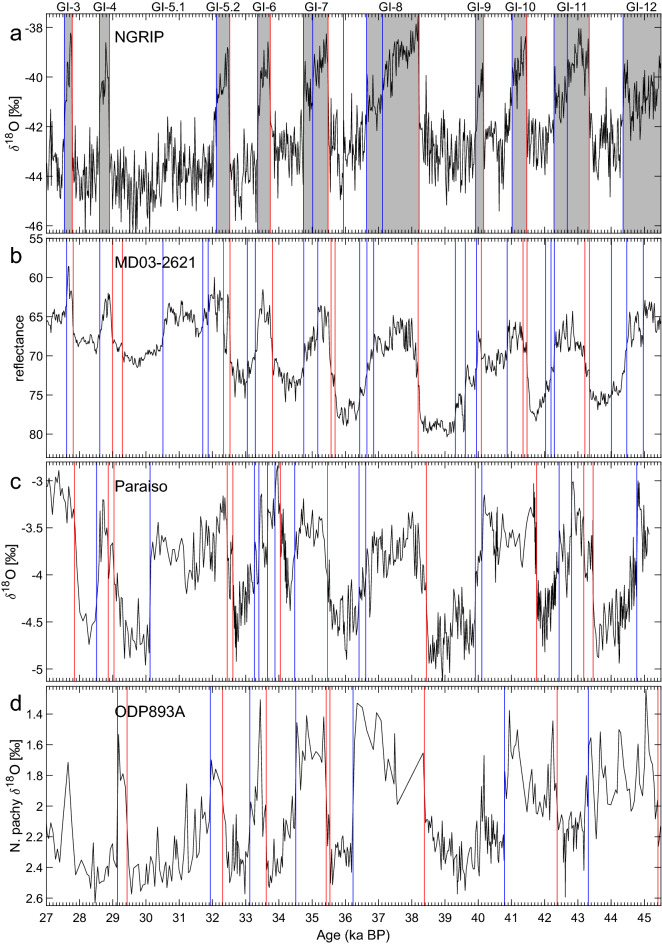
Comparison of four paleorecords over the 45.5–27 ka BP interval: (**a**) NGRIP ice core $\delta ^{18}$O^131^; (**b**) MD03-2621 marine sediment reflectance^64^; (**c**) Paraiso Cave (PAR07) $\delta ^{18}$O^165^; and (**d**) ODP893A marine sediment *N. pachyderma* $\delta ^{18}$O^87,88^. Vertical lines represent detected transitions, with colors indicating the direction of the jumps. Grey bars in panel (**a**) represent interstadials identified for the NGRIP $\delta ^{18}$O record by Bagniewski et al.^19^. Note that the “ka BP” time unit, where the ‘Present’ is defined as the year 1950, applies only to the Paraiso and ODP893A records. The NGRIP and MD03-2621 records use the “ka b2k” time scale, where ‘b2k’ refers to the year 2000 as the origin of past times. The vertical axes in (**b, d**) are reversed.

In Fig. 6, we compare four paleorecords of different types and from distinct locations: NGRIP $\delta ^{18}$O, MD03-2621 reflectance, Paraiso Cave $\delta ^{18}$O, and ODP893A planktic $\delta ^{18}$O from the Santa Barbara Basin. Overall, the abrupt transitions in the four records appear to be fairly synchronous, with the main Greenland DO events from NGRIP also observed for the two marine records and the one speleothem record. The transitions that correspond to Greenland interstadials (GIs) GI-12 to GI-3^131^ are identified in all four records, with only a few exceptions: GI-3 is not identified for the ODP893A record, due to an insufficient number of data points; and GI-5.1 is not identified in any record, except as a cooling event in MD03-2621 and in Paraiso cave. Also, GI-10 and GI-9 appear as a single interstadial in the Paraiso cave and ODP893A records, which is probably due to a decreased time resolution observed in both records during this time interval.

**Figure 7:**
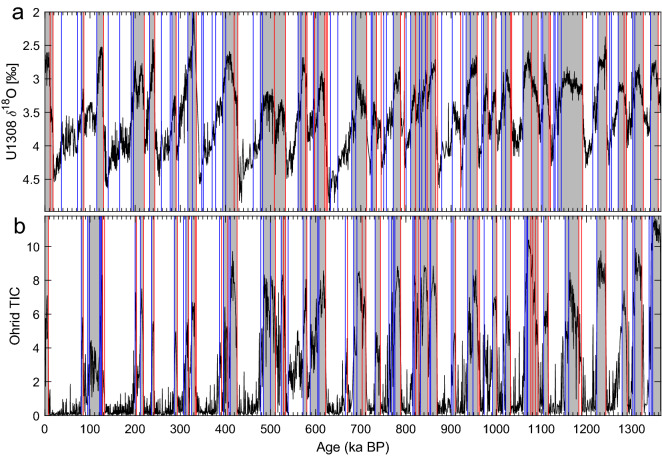
Comparison of the benthic *Cibicidoides sp.* $\delta ^{18}$O record in (**a**) the U1308 marine-sediment core^95^ and (**b**) Lake Ohrid TIC^137^. Vertical lines represent transitions detected by our KS methodology^19^, with colors indicating the direction of the jumps. Grey bars correspond to interglacials (odd-numbered MISs) and white bars correspond to glacials (even-numbered MISs). The vertical axis in (**a**) is reversed.

Finally, Fig. 7 shows the comparison between the U1308 benthic $\delta ^{18}$O record and the Lake Ohrid Total Inorganic Carbon (TIC) record over a 1.4 Myr time interval that includes multiple glacial-interglacial transitions.

The U1308 benthic $\delta ^{18}$O record in panel (a) of the figure is a 3.1 Myr record located within the ice-rafted detritus belt of the North Atlantic^196^. This record is a proxy for deep-water temperature and global ice volume, and it has enabled a detailed reconstruction of the history of orbital and millennial-scale climate variability during the Quaternary; it documents the changes in Northern Hemisphere ice sheets that follow the glacial–interglacial cycles, as well as mode transitions identified at 2.55 Ma, 1.5 Ma, 1.25 Ma, 0.65 Ma and 0.35 Ma BP^95,197^. Here, we only show the 1.4–0 Ma BP time interval that corresponds to the length of the Lake Ohrid record. For the full 3.1 Myr U1308 record, please see Fig. S2 in the Supplementary Information.

The 1.25–0.65 Ma BP interval in the figure corresponds to the Mid-Pleistocene Transition, characterized by an increase in the amplitude of the glacial–interglacial fluctuations and a shift from a predominantly 40 kyr to a predominantly 100 kyr periodicity; see Reichers et al.^185^ and references therein. The abrupt transition at 0.35 Ma marks the start of the strongest interglacial of the record, which corresponds to the marine isotope stage (MIS) 9. Here, our KS analysis agrees with the well established marine isotope stratigraphy of Lisiecki and Raymo^198^ by detecting rapid warmings that correspond to the classical terminations leading to interglacials, as well as rapid coolings that initiate glacial stages.

The Lake Ohrid TIC record in Fig. 7b shows glacial–interglacial variability in biomass over the past 1.4 Myr. Here, our KS analysis shows numerous abrupt transitions towards high TIC intervals that correspond to interglacial episodes, as well as matching ones of opposite sign. The interglacials are associated with forested environmental conditions that are consistent with odd-numbered MISs, as was the case for the U1308 record in the figure’s panel (a). The glacial episodes, to the contrary, correspond to forestless conditions, that, according to the available timescale, are consistent with even-numbered MISs.

## Discussion

### The two methods

The augmented KS test of Bagniewski et al.^19^ has detected abrupt transitions on different time scales in a variety of paleorecords. These include the NGRIP ice core, the Paraiso Cave speleothem, and the MD03-2621 and ODP893A marine sediment cores for the last glacial cycle; the U1308 marine sediment core and Lake Ohrid TIC for the Middle and Late Pleistocene; and the CENOGRID marine sediment stack for the Cenozoic Era. While possible mechanisms giving rise to the observed variability in these records have been discussed in previous studies, the objective, precise and robust dating of the main transitions using the augmented KS test may allow a more detailed and definitive analysis and modeling.

The transitions identified for the MD03-2621 reflectance record with the RQA methodology^20,21,180^ do correspond to a subset of those found with the KS test, but several smaller-scale transitions identified by the latter have not been found with the former method. As discussed in Bagniewski et al.^19^, recognizing the transition points by RQA becomes increasingly difficult at time scales shorter than the window length. The RQA approach, though, does allow one to quantify the magnitude of each transition, and it may thus be useful for identifying key transitions. Moreover, RPs may be helpful in illustrating changes in periodicity.

### Interpretation of findings

The high-resolution MD03-2621 reflectance record from the Cariaco Basin^64^ in Fig. 2 shows abrupt transitions that are largely in agreement with those detected by our KS test for the NGRIP record. Desplazes et al.^64^ have argued that these transitions are driven by the ITCZ displacements that occurred primarily in response to Northern Hemisphere temperature variations. These authors indicated that the ITCZ migrated seasonally during mild stadials, but was permanently displaced south of the Cariaco Basin during the colder stadial conditions. The very high resolution of the MD03-2621 record allows a detailed comparison with the transitions identified for the NGRIP record. The fact that several small-scale NGRIP transitions are not identified by our KS test in the MD03-2621 record suggests that a TP linked to ITCZ migration was not crossed during these events. Furthermore, the KS test does reveal transitions that have not been recognized previously in either record^131^, e.g., at 86.15 ka BP, which we find in both the NGRIP and MD03-2621 records.

In the 67-Myr CENOGRID stack (Fig. 4) of benthic $\delta ^{18}$O^23,187^, we identified four major cooling transitions that culminate with the start of the Pleistocene, as well as two warming transitions, including the PETM. These major jumps agree with those identified in Westerhold et al.^23^. However, using a shorter window length, many more transitions were detected during the Pleistocene, well known for its higher climate variability^6,194^, as well as during the early Miocene, i.e., between the Oligocene-Miocene transition at 23 Ma BP^199–201^ and the mid-Miocene transition at 14 Ma BP^191^. A possible reason is the fact that the 20–13 Ma BP interval in the stack has been constructed using records from the eastern Tropical Pacific. This region has been characterized by high upwelling rates and sedimentation rates in the past^202^. While the CENOGRID composite has a uniform resolution, higher sedimentation rates could affect the resolution of the original records and, therewith, even the variability seen in lower-resolution sampling.

It follows from these remarks on Fig. 4a, b that variations in resolution of the data, which can arise from various factors including measurement techniques and source region characteristics, can have a considerable effect on the frequency of detected transitions. These factors can significantly impact the reliability of transition detection methods, thus highlighting the need for caution when using individual records as proxies for global climate. While statistical indicators allow us to quantify jumps in the data without any climatic context being provided, it is important to consider the limitations of such an approach, and to supplement it with an independent understanding of the proxy data whenever possible. By doing so, we can better contextualize and understand the implications of these jumps.

The Paraiso Cave record^165^ from the eastern Amazon lowlands in Fig. 5 shows drier conditions during interglacials, with abrupt transitions matching those that correspond to the DO events identified in the NGRIP ice core record. The Amazon record is also in fairly good agreement with the nearby MD03-2621 marine sediment record. Notably, several of NGRIP’s DO events appear combined in both records, namely GI-5.2 and GI-5.1, as well as GI-10 and GI-9, which might indicate that a climate change event over Greenland did not trigger a tipping event in the Amazon basin. Alternatively, the first merging may question the separation of the classical GI-5 event^203^ into two distinct events, 5.2 and 5.1.

The comparison of four records on the same time scale in Fig. 6 demonstrates that a signal of abrupt climate change is detected when using the KS method with similar accuracy for different types of paleodata. The differences in the dates of the transitions may be largely explained by the use of different age models in each of the records, with MD03-2621 fine-tuned to the NGRIP chronology, and ODP893A fine-tuned to the GISP2 chronology, while the NGRIP and Paraiso Cave records were independently dated. Notably, ODP893A data prior to GI-8 appear misaligned with the other three records. The chronology of jumps in ODP893A indicates that the warm interval between 42.4 ka BP and 40.8 ka BP may correspond to an event spanning GI-10 and GI-9, while the warm interval between 45.3 ka BP and 43.3 ka BP may correspond to GI-11.

It is important to keep in mind that age estimates of many paleoclimate records, including some of those discussed here, are often not determined through independent methods, but rather are “tuned” or wiggle-matched to other records, such as the global benthic $\delta ^{18}$O stack^198^ or the NGRIP $\delta ^{18}$O record^131^. As the abrupt jumps in these records are themselves used as tie-points, it is expected that the KS test would show a similarity in the timing of jumps in records that were tuned to the same reference record. Therefore, any differences in the timing of transitions between these records should not be taken as indicative of the chronology of events, but rather as a result of differences between the KS method and the transition detection method used in the wiggle matching process. This fact should be taken into consideration when comparing records that have not been independently dated.

In addition to the classical GI transitions, several additional jumps are identified in each of the marine and cave records. These jumps may be the representative of local climate changes or, in some cases, be artifacts of sampling resolution or measurement error. Stronger evidence for a local or regional event may be obtained when comparing two nearby records, like the Paraiso Cave in the Amazon and the MD03-2621 marine record from the Cariaco Basin. In both records, the start of GI-5.2 is represented as two warming transitions, in contrast with the NGRIP and ODP893A records, where only one sharp transition is present. Likewise, the end of GI-9 in the two tropical, South American records appears as two successive cooling events. These results point to the potential of using the KS method to improve fine-tuning the synchroneity of two or more records, when an independent dating method is not available.

The comparison in Fig. 7 of KS-detected transitions in marine core U1308^95^ and in Lake Ohrid TIC^137^ identifies the transitions between individual glacials and interglacials. In the two records, the transitions are well identified, despite the resolution of the two being different, and they offer a precise dating for the chronology of the past glacial cycles. The transitions are overall in good agreement between the two records. Still, the warm events in Lake Ohrid are essentially atmospheric and thus have often a shorter duration than in the marine record, while cooling transitions precede those in the deep ocean by several thousand years. This could indicate that a significant time lag is present, either in ice sheet growth in response to atmospheric cooling, or in the propagation of the cooling signal into the deep North Atlantic.

Furthermore, there are atmospheric interglacial episodes missing in the oceanic U1308 record. This mismatch between the lake record^137^ and the marine one^95^ could be attributed to the fact that the KS test does sometimes find more transitions in one record than in another one, even for the same type of proxy and within the same region. Such occurrences may be due to the local environment, the sampling method, or some aspect of the KS method itself. When this is the case, it is important—although not always feasible—to obtain additional records covering a similar time interval with a similar resolution, in order to shed further light on the mismatch between the two original records. It should be noted that this study analyzed only one proxy type from each location. A more comprehensive representation of past transitions could be achieved by investigating different proxy records from the same core. For example, in contrast to the Rasmussen et al.^131^ study of the NGRIP record, which analyzed both the $\delta ^{18}$O and Ca$^{2+}$ proxies, this study only utilized $\delta ^{18}$O (Fig. 6a), which is a likely reason for not detecting the GI-5.1 event.

## Concluding remarks

The carefully selected, high-quality paleoproxy records in the open-source PaleoJump database^18^, https://paleojump.github.io, have different temporal scales and a global spatial coverage; see again Fig. 1. These records provide an easily accessible resource for research on potential tipping elements in Earth’s climate. Still, major gaps in the marine sediment records exist in the Southern Hemisphere, especially in the Indian and Pacific Oceans. Only sparse terrestrial data are available from the high latitudes in both hemispheres, due to the recent glaciation. The only data from the African continent come from two East African lake sediment records. Even though much information is at hand from the more than 100 sites listed in this paper, more records are needed to fill geographical and temporal gaps, especially in the Southern Hemisphere.

The examples given in the paper on abrupt-transition identification demonstrate the usefulness of the records included in PaleoJump for learning about potential tipping events in Earth’s history and for comparing such events across different locations around the world. The accessibility of such high-quality records is an invaluable resource for the climate modeling community that requires comparing their results across a hierarchy of models^184,204^ with observations.

We also demonstrated the usefulness of the KS test^19^ for reconstructing the chronology of Earth’s main climatic events. The newly developed tool for automatic detection of abrupt transitions may be applied to different types of paleorecords, allowing to objectively and robustly characterize the tipping phenomenon for climate subsystems already suspected of being subject to tipping^9^, but also to identify previously unrecognized tipping elements in past climates. The observational descriptions of tipping that can be obtained from PaleoJump using our KS methodology, combined with the application of Earth System Models, can help improve the understanding of the bifurcation mechanisms of global and regional climate and identify possible TPs for future climates.

Our results also indicate that paleorecord interpretations may vary, since the abrupt transitions identified in them will depend on the time scale and type of variability that is investigated. For example, the KS method’s parameters^19^ may be changed when studying different proxy record resolutions, affecting the frequency and exact timing of the TPs that are identified.

The agreement in timing and pattern between jumps in distinct records can confirm the correctness of each record separately, as well as of the inferences on climate variability drawn from these jumps. Specifically, the ability of the KS method to identify matching small-scale transitions in different high-resolution records may be used to validate these transitions as being the result of genuine global or regional climatic events, as opposed to just sampling errors. Furthermore, significant differences in records that are, overall, in good agreement with each other may help decode the chronology of tipping events or an approximate range for a tipping threshold. A fortiori, the differences in timing and pattern between jumps in distinct records that we also found emphasize the importance of a consistent dating methodology.

In a potential second step, the transition detection tools presented here could be used for harmonizing and synchronizing the records of the PaleoJump database, in a way that resembles the work conducted by several research teams in recent years^13,159,205–208^. Doing so would facilitate interpretating the abrupt changes in these records.

The broad spatial coverage of the PaleoJump database^18^, https://paleojump.github.io, with its records that vary in their nature—ice, marine and land—as well as in their length and resolution, will facilitate research on tipping elements in Earth’s climate, including the polar ice sheets, the Atlantic Meridional Overturning Circulation, and the tropical rainforests and monsoon systems. Furthermore, it will support establishing improved criteria on where and how to collect data for reliable early warning signals of impending TPs.

## Supplementary Information

Supplementary Information.

## Data Availability

The datasets analyzed during the current study are available through Zenodo (https://doi.org/10.5281/zenodo.6534031)^18^ and on the PaleoJump website, (https://paleojump.github.io). The source code of the PaleoJump website can be found on GitHub at (https://github.com/paleojump/paleojump). The codes used for KS and RQA analyses are part of the TiPES statistical toolbox, available on GitHub at (https://github.com/paleojump/TiPES_statistical_toolbox). The ETOPO1 Global Relief Model data^175^ used for making Fig. 1 is available from NOAA at (https://www.ncei.noaa.gov/products/etopo-global-relief-model).
