# Supplementary material for: The PaleoJump database for abrupt transitions in past climates
Source: Sci Rep. 2023 Mar 18;13:4472. doi: 10.1038/s41598-023-30592-1 (PMC10024733; doi:10.1038/s41598-023-30592-1)
Supplement: Supplementary file 1 — Supplementary Information. [file 41598_2023_30592_MOESM1_ESM.pdf]

Supplementary Information for: The PaleoJump database for abrupt transitions in past climates

Witold Bagniewski[1,*], **Denis-Didier Rousseau**[2,3,4], **and Michael Ghil**[1,5]

[1]Department of Geosciences and Laboratoire de Météorologie Dynamique (CNRS and IPSL), École Normale Supérieure and PSL University, Paris, France
[2]Geosciences Montpellier, University of Montpellier, CNRS, Montpellier, France
[3]Institute of Physics - CSE, Division of Geochronology and Environmental Isotopes, Silesian University of Technology, Gliwice, Poland
[4]Lamont-Doherty Earth Observatory, Columbia University, New York, USA
[5]Department of Atmospheric and Oceanic Sciences, University of California at Los Angeles, Los Angeles, USA
[*]wbagniewski@lmd.ipsl.fr

ABSTRACT

Tipping points (TPs) in Earth's climate system have been the subject of increasing interest and concern in recent years, given the risk that anthropogenic forcing could cause abrupt, potentially irreversible, climate transitions. Paleoclimate records are essential for identifying past TPs and for gaining a thorough understanding of the underlying nonlinearities and bifurcation mechanisms. However, the quality, resolution, and reliability of these records can vary, making it important to carefully select the ones that provide the most accurate representation of past climates. Moreover, as paleoclimate time series vary in their origin, time spans, and periodicities, an objective, automated methodology is crucial for identifying and comparing TPs. To address these challenges, we introduce the open-source PaleoJump database, which contains a collection of carefully selected, high-resolution records originating in ice cores, marine sediments, speleothems, terrestrial records, and lake sediments. These records describe climate variability on centennial, millennial and longer time scales and cover all the continents and ocean basins. We provide an overview of their spatial distribution and discuss the gaps in coverage. Our statistical methodology includes an augmented Kolmogorov-Smirnov test and Recurrence Quantification Analysis; it is applied here, for illustration purposes, to selected records in which abrupt transitions are automatically detected and the presence of potential tipping elements is investigated. These transitions are shown in the PaleoJump database along with other essential information about the records, including location, temporal scale and resolution, as well as temporal plots. This open-source database represents, therefore, a valuable resource for researchers investigating TPs in past climates.

Supplementary Tables

The sites of proxy records included in the PaleoJump database have been compiled in five tables according to their geological nature. For each site, the available data have been analyzed to determine the essential information, which is given in the tables below: location, depth/elevation, temporal range, maximum temporal resolution, and types of paleoproxies. This information is accompanied by links to the original data and the associated publications. The “maximum resolution” value in the tables is calculated as the maximum of the average temporal resolution for a 10-kyr time interval, excluding the Holocene and the late deglacial, i.e. the last 14 000 years, during which the proxy time resolution is frequently much higher than for the older part of the record. This was done so as to allow a more accurate comparison of the centennial- and millennial-scale variabilities between different records of the glacial and earlier interglacials periods.

1. Records included in the PaleoJump database

Site name	Location	Depth	Age	Res.	Proxies
MD95-2010[1]	66.684, 4.566	1226 m	67 - 10 ka	35 y	pla δ^{18}O; ben δ^{18}O; pla δ^{13}C; ben δ^{13}C; IRD; mag sus
ODP162-983[2]	60.403, -23.641	1984 m	1.2 - 0 Ma	89 y	IRD; %NPS
SO82-5[3,4]	59.186, -30.905	1416 m	57 - 15 ka	62 y	pla δ^{18}O; ben δ^{18}O; pla δ^{13}C; ben δ^{13}C; SST; IRD
MD95-2006[5]	57.03, -10.058	2122 m	56 - 40 ka	90 y	pla δ^{18}O; ben δ^{18}O; pla δ^{13}C; ben δ^{13}C; %NPS; SST; IRD
JPC-13[6]	53.057, -33.53	3082 m	128 - 7 ka	12 y	pla δ^{18}O; pla δ^{13}C; ben δ^{18}O; ben δ^{13}C; grain size; XRF
U1308[7,8]	49.878, -24.238	3871 m	3.14 - 0 Ma	118 y	ben δ^{18}O; ben δ^{13}C; bulk carbonate δ^{18}O; Ca/Sr; Si/Sr; pla δ^{18}O
MD01-2412[9]	44.523, 145.003	1225 m	116 - 0 ka	113 y	SST
MD99-2331[10–13]	42.15, -9.683	2120 m	160 - 16 ka	137 y	pla δ^{18}O; IRD; SST; pollen; temperate forest pollen
U1313[14–16]	41, -32.957	3426 m	4.3 - 0 Ma	185 y	pla δ^{18}O; ben δ^{18}O; SST; Qz/Cal
MD95-2040[17,18]	40.582, -9.861	2465 m	360 - 0 ka	78 y	pla δ^{18}O; pla δ^{13}C; ben δ^{18}O; ben δ^{13}C; SST; IRD
MD95-2039[10,19]	40.579, -10.349	3381 m	51 - 0 ka	107 y	pla δ^{18}O; IRD; SST
MD01-2443[17,20]	37.881, -10.176	2925 m	433 - 86 ka	188 y	pla δ^{18}O; ben δ^{18}O; ben δ^{13}C; SST
MD95-2042[10,11,21,22]	37.8, -10.167	3146 m	418 - 0 ka	83 y	pla δ^{18}O; ben δ^{18}O; ben δ^{13}C; pla δ^{13}C; SST
U1385[23,24]	37.571, -10.126	2587 m	1.4 - 0 Ma	47 y	pla δ^{18}O; reflectance; ben δ^{18}O; SST
MD01-2444[20,21]	37.565, -10.134	2656 m	420 - 0 ka	86 y	pla δ^{18}O; ben δ^{18}O; ben δ^{13}C; reflectance; SST
MD99-2341[10]	36.389, -7.066	582 m	49 - 1 ka	104 y	pla δ^{18}O
ODP977a[21]	36.032, -1.955	1984 m	244 - 0 ka	181 y	SST
MD99-2339[19,25]	35.886, -7.528	1177 m	47 - 0 ka	38 y	pla δ^{18}O; ben δ^{18}O; pla δ^{13}C; ben δ^{13}C; SST; grain size
M40/4_SL71[26–28]	34.811, 23.194	2788 m	182 - 0 ka		grain size; XRF; clay; pla δ^{18}O
ODP893A[29–31]	34.28, -120.03	576 m	65 - 0 ka	41 y	pla δ^{18}O; pla δ^{13}C; ben δ^{18}O
U1429[32]	31.617, 128.998	732 m	393 - 0 ka		pla δ^{18}O; ben δ^{18}O; ben δ^{13}C; pla δ^{13}C; SST
MD02-2575[33]	29.002, -87.119	847 m	400 - 1 ka	135 y	pla δ^{18}O; ben δ^{18}O; SST
MD04-2876[34]	24.843, 64.008	828 m	50 - 0 ka	169 y	δ^{15}N; total N; TOC
SO90-93KL[35]	23.583, 64.217	1802 m	109 - 1 ka	177 y	pla δ^{18}O
SO130-289KL[36,37]	23.122, 66.497	571 m	79 - 2 ka	0.2 y	reflectance; grain size; TOC; ...
SO90-136KL[35]	23.117, 66.5	568 m	66 - 2 ka	63 y	TOC
SO90-111KL[35]	23.1, 66.483	775 m	62 - 2 ka	72 y	TOC
ODP658C[38]	20.75, -18.583	2263 m	84 - 0 ka	135 y	SST
SO188-17286-1[39]	19.743, 89.879	1428 m	129 - 0 ka	158 y	pla δ^{18}O; ben δ^{18}O; SST
U1446[40]	19.083, 85.733	1440 m	1.46 - 0 Ma	24 y	Rb/Ca
GeoB9526-5[41,42]	12.435, -18.057	3223 m	72 - 1 ka	55 y	Fe/K; SST; ben δ^{18}O
NIOP905[43]	10.767, 51.951	1580 m	88 - 1 ka	145 y	ben δ^{18}O; ben δ^{13}C
MD03-2621[36]	10.678, -64.972	847 m	109 - 6 ka	0.1 y	reflectance
MD97-2141[44]	8.78, 121.28	3633 m	395 - 5 ka	51 y	pla δ^{18}O
MD98-2181[45,46]	6.3, 125.83	2114 m	68 - 0 ka	66 y	pla δ^{18}O; pla δ^{13}C; ben δ^{18}O; SST
MD03-2707[47]	2.502, 9.395	1295 m	155 - 0 ka	92 y	pla δ^{18}O; SST; BWT
TR163-22[48]	0.52, -92.4	2830 m	135 - 1 ka	169 y	pla δ^{18}O; ben δ^{18}O; SST
SO189-039KL[49]	-0.79, 99.908	517 m	45 - 0 ka	66 y	pla δ^{18}O; SST

Site name	Location	Depth	Age	Res.	Proxies
GeoB6518-1[50,51]	-5.588, 11.222	962 m	43 - 0 ka		pla δ^{18}O; ben δ^{18}O; TOC; SST
GeoB7139-2[52]	-30.2, -71.983	3267 m	70 - 1 ka	53 y	δ^{15}N; total N
MD03-2611G[53]	-36.73, 136.548	2420 m	94 - 0 ka	15 y	pla δ^{18}O; pla δ^{13}C; SST; quartz; Ti
ODP1089[54]	-40.93, 9.9	4621 m	615 - 0 ka	189 y	pla δ^{18}O; ben δ^{18}O; pla δ^{13}C; ben δ^{13}C
TNO57-21[55]	-41.1, 7.8	4981 m	99 - 0 ka	112 y	pla δ^{18}O; %NPS
MD02-2588[56]	-41.332, 25.828	2907 m	353 - 0 ka	246 y	ben δ^{18}O; ben δ^{13}C; pla δ^{13}C
ODP181-1123[57]	-41.786, -171.499	3290 m	1.5 - 0 Ma	388 y	ben δ^{18}O; ben δ^{13}C; Mg/Ca
MD07-3076Q[58,59]	-44.153, -14.228	3770 m	67 - 1 ka	61 y	pla δ^{18}O; pla δ^{13}C; SST
MD97-2120[60]	-45.534, 174.931	1210 m	151 - 4 ka	103 y	pla δ^{18}O; SST
MD88-770[61]	-46.017, 96.467	3290 m	149 - 5 ka	187 y	SST
CENOGRID stack[62]	N/A	N/A	67.1 - 0 Ma	2000 y	ben δ^{18}O; ben δ^{13}C

Supplementary Table S 1. Marine Sediment Cores, ordered by latitude. Res.: temporal resolution, pla: planktic, ben: benthic, SST: sea surface temperature, BWT: bottom water temperature, IRD: ice rafted detritus, TOC: total organic carbon, mag sus: magnetic susceptibility.

Site name	Location	Elevation	Age	Res.	Proxies
NEEM[63–65]	77.45, -51.06	2545 m	129 - 0 ka	4 y	δ^{18}O; Ca^{2+}; Na+; ...
NGRIP[66,67]	75.1, -42.32	2925 m	122 - 0 ka	20 y	δ^{18}O; Ca; dust; CH4; ...
GISP2[66]	72.97, -38.8	3208 m	104 - 0 ka	20 y	δ^{18}O; Ca^{2+}; CH4; δ^{15}N; ...
GRIP[66]	72.58, -37.63	3200 m	104 - 0 ka	20 y	δ^{18}O; Ca; CH4; ...
Guliya[68]	35.28, 81.48	6200 m	132 - 0 ka	400 y	δ^{18}O; dust; ...
TALDICE[69,70]	-72.783, 159.067	2315 m	314 - 0 ka	39 y	δ^{18}O; CH4; Fe
EPICA EDML[71]	-75.003, 0.068	2416 m	150 - 0 ka	19 y	δ^{18}O
EPICA Dome C[72–77]	-75.1, 123.35	3189 m	802 - 0 ka	39 y	δD; δT; CO2; CH4; dust; δ^{18}O; Ca^{2+}
Dome Fuji[78]	-77.32, 38.7	3810 m	716 - 0 ka	34 y	δ^{18}O; dust
Vostok[79]	-78.47, 106.8	3488 m	423 - 0 ka	55 y	δD; δT; CH4; dust; ...
WAIS Divide[80]	-79.468, -112.087	1806 m	68 - 0 ka	12 y	δ^{18}O; CH4; ...
Synthetic Greenland[81]	N/A	2135 m	798 - 5 ka	50 y	δ^{18}O

Supplementary Table S 2. Ice cores, ordered by latitude.

Site name	Location	Elevation	Age	Res.	Proxies
Gassel Tropfsteinhöhle Cave[82]	47.823, 13.843	1225 m	108 - 77 ka	5 y	δ^{18}O
Grete-Ruth Cave[82]	47.543, 12.027	1435 m	111 - 103 ka	12 y	δ^{18}O
Hölloch im Mahdtal Cave[82]	47.378, 10.151	1438 m	74.4 - 73.6 ka	5 y	δ^{18}O
Schneckenloch Cave[82,83]	47.375, 10.068	1285 m	118 - 64 ka	7 y	δ^{18}O
Grosser Baschg Cave[82,83]	47.25, 9.667	785 m	87 - 81 ka	14 y	δ^{18}O
Villars Cave (Vil-stm09)[84,85]	45.442, 0.785	175 m	83 - 31 ka	88 y	δ^{18}O; δ^{13}C
Kesang Cave[86]	42.93, 81.78	2000 m	500 - 52 ka	23 y	δ^{18}O
Sofular Cave[87]	41.416, 31.934	700 m	50 - 0 ka	18 y	δ^{18}O; δ^{13}C
Tonnel'naya Cave[88]	38.4, 67.23	3226 m	134 - 8 ka	1 y	δ^{18}O; δ^{13}C
Leviathan Cave[89]	37.831, -115.607	2400 m	174 - 0 ka	50 y	δ^{18}O
Dim Cave (Dim-E3)[90]	36.54, 32.109	232 m	90 - 10 ka	82 y	δ^{18}O; δ^{13}C
Devils Hole[91]	36.425, -116.291	719 m	204 - 5 ka	65 y	δ^{18}O; δ^{13}C
Fort Stanton Cave[92]	33.507, -105.494	1864 m	56 - 11 ka	23 y	δ^{18}O
Hulu Cave (MSD; MSL)[93]	32.058,119.041	86 m	76 - 18 ka	68 y	δ^{18}O; U/Th ages
Cave of the Bells[94]	31.729, -110.768	1639 m	53 - 11 ka	18 y	δ^{18}O
Sanbao Cave[95,96]	31.667, 110.433	1900 m	641 - 0 ka		δ^{18}O; U/Th ages

Site name	Location	Elevation	Age	Res.	Proxies
Soreq Cave[97]	31.45, 35.03	400 m	184 - 0 ka	44 y	δ^{18}O; δ^{13}C
Bittoo Cave[98]	30.79, 77.776	3000 m	284 - 0 ka	18 y	δ^{18}O; δ^{13}C
Abaco Island Cave[99]	26.23, -77.16	-17 m	64 - 14 ka	14 y	δ^{18}O; δ^{13}C; Sr/Ca; Mg/Ca
Dongge Cave[100]	25.28, 108.08	680 m	146 - 99 ka	20 y	δ^{18}O; U/Th ages
Moomi Cave[101]	12.533,54.317	400 m	53 - 40 ka	15 y	δ^{18}O
Terciopelo Cave[102]	10.167, -85.333	370 m	98 - 24 ka	100 y	δ^{18}O
Northern Borneo[103]	4.085, 114.85	250 m	162 - 0 ka	55 y	δ^{18}O
Santiago Cave[104]	-3.017, -78.133	980 m	94 - 6 ka	49 y	δ^{18}O
Paraiso Cave (PAR07)[105]	-4.067, -55.45	60 m	45 - 18 ka	21 y	δ^{18}O; δ^{13}C
Pacupahuain Cave[106]	-11.24, -75.82	3800 m	50 - 16 ka	27 y	δ^{18}O; δ^{13}C
Lapa Sem Fim Cave[107]	-16.149, -44.627	690 m	84 - 12 ka	8 y	δ^{18}O
Lapa Grande Cave[107]	-16.707,-43.943	730 m	84 - 40 ka	14 y	δ^{18}O
Ball Gown Cave[108]	-17.28, 125.12	100 m	40 - 8 ka		δ^{18}O
Botuvera Cave[109]	-27.225, -49.158	230 m	116 - 0 ka	95 y	δ^{18}O; δ^{13}C
Hollywood Cave[110]	-41.952, 171.476	130 m	73 - 11 ka	58 y	δ^{18}O; δ^{13}C
China cave composite[96]	N/A	N/A	641 - 0 ka	22 y	δ^{18}O; U/Th ages

Supplementary Table S 3. Speleothems, ordered by latitude.

Site name	Location	Elevation	Age	Res.	Proxies
ELSA-Eifel loess stack[111]	50.16, 6.83	403 m	132 - 0 ka	100 y	dust
Nussloch loess[112,113]	49.316, 8.722	180 m	130 - 18 ka		grain size; δ^{13}C; Snails; earth worms; mag sus; paleosol-loess doublets
Dunaszekcso loess[114]	46.09, 18.763	135 m	150 - 20 ka		grain size; paleosol-loess doublets
Chashmanigar loess	38.392, 69.833	1400 m	1.77 - 0 Ma		grain size; mag sus; reflectance
Gulang loess[115–117]	37.49, 102.88	2400 m	1.48 - 0 Ma	50 y	grain size; δ^{13}C
Jiyuan loess[118]	37.14, 107.39	1730 m	130 - 0 ka		grain size
Zichang loess[118]	37.14, 109.85	1265 m	249 - 0 ka		grain size
Hongde loess[118]	36.77, 107.21	1640 m	249 - 0 ka		grain size
Huanxian loess[118]	36.65, 107.26	1500 m	249 - 0 ka		grain size
Jingyuan loess[115–117,119]	36.35, 104.62	2050 m	1.7 - 0 Ma	26 y	grain size; δ^{13}C
Huachi loess[118]	36.34, 107.93	1395 m	249 - 0 ka		grain size
Xinzhuangyuan loess[118]	36.19, 104.73	2110 m	249 - 0 ka		grain size
Linxia loess[117,118]	36.15, 103.63	2210 m	660 - 0 ka		grain size
Lijiayuan loess[118]	36.12, 104.86	1850 m	249 - 0 ka		grain size
Yimaguan loess[120]	35.917, 107.617	1500 m	879 - 0 ka	197 y	grain size; mag sus
Luochuan loess[120]	35.717, 109.417	1100 m	884 - 0 ka	289 y	grain size; mag sus
Chinese Loess Plateau stack[121]	35.41,107.73	1300 m	3.6 - 0 Ma	1000 y	grain size; mag sus
Chiloparts loess stack[122]	35.3, 107.6	1300 m	2.6 - 0 Ma		grain size
Lingtai loess[119]	35.067, 107.65	1350 m	7 - 0 Ma	107 y	grain size; mag sus; carbonate
CHILOMOS loess stack[118]	N/A	N/A	249 - 0 ka	200 y	grain size

Supplementary Table S 4. Loess and dust records, ordered by latitude.

Site name	Location	Elevation	Age	Res.	Proxies
Lake El'gygytgyn[123,124]	67.5, 172.104	489 m	3.6 - 0 Ma	27 y	Mn/Fe; Si/Ti; mag sus; TOC; TIC; biogenic Si
Lake Baikal[125]	53.696, 108.352	456 m	1.8 - 0 Ma	223 y	biogenic Si
Füramoos[126]	47.983, 9.883	662 m	140 - 0 ka		pollen
Les Echets[127,128]	45.833, 5	267 m	46 - 15 ka		pollen; diatoms; mag sus; geochemistry

Site name	Location	Elevation	Age	Res.	Proxies
Lac du Bouchet[13,129]	44.83, 3.82	1200 m	70 - 0 ka	89 y	pollen; temperate forest pollen
Summer Lake[130]	42.83, -120.75	1260 m	46 - 23 ka	76 y	$\delta^{18}O$; $\delta^{13}C$
Valle di Castiglione[13,131]	41.9, 12.76	44 m	56 - 14 ka	357 y	pollen; temperate forest pollen
Tenaghi Philippon[132]	41.17, 24.33	40 m	1.35 - 0 Ma		pollen
Lake Ohrid[133–136]	41.049, 20.715	693 m	1.36 - 0 Ma	208 y	$\delta^{18}O$; $\delta^{13}C$; TIC; Zr/K; pollen; ...
Lago di Monticchio[137–139]	40.932, 15.605	656 m	100 - 10 ka	120 y	pollen; temperate forest pollen; biogenic Si; dry density
Ioannina[13,140]	39.75, 20.85	470 m	130 - 0 ka	150 y	pollen; temperate forest pollen
Lake Van[141]	38.667, 42.669	1649 m	250 - 129 ka	336 y	$\delta^{18}O$; $\delta^{13}C$; pollen; ...
Padul[142–144]	37, -3.67	785 m	197 - 0 ka	102 y	pollen, precipitation
Dead Sea[145]	31.508, 35.471	-428 m	88 - 14 ka	242 y	pollen
Lake Tulane[146]	27.584, -81.502	36 m	61 - 0 ka		Pinus pollen
Lake Tanganyika[147]	-6.714, 29.833	773 m	59 - 1 ka	260 y	$\delta^{13}C$; δD; Lake Surface Temperature
Lake Malawi[148]	-11.294, 34.437	500 m	1.28 - 0 Ma	14 y	Ca; T; $\delta^{13}C$
Lake Titicaca[149,150]	-15.937, -69.16	3810 m	370 - 3 ka	28 y	TOC; $\delta^{13}C$; grain size

Supplementary Table S 5. Lake sediment cores, ordered by latitude.

Supplementary Figures

CENOGRID stack, including transitions detected using a shorter window length

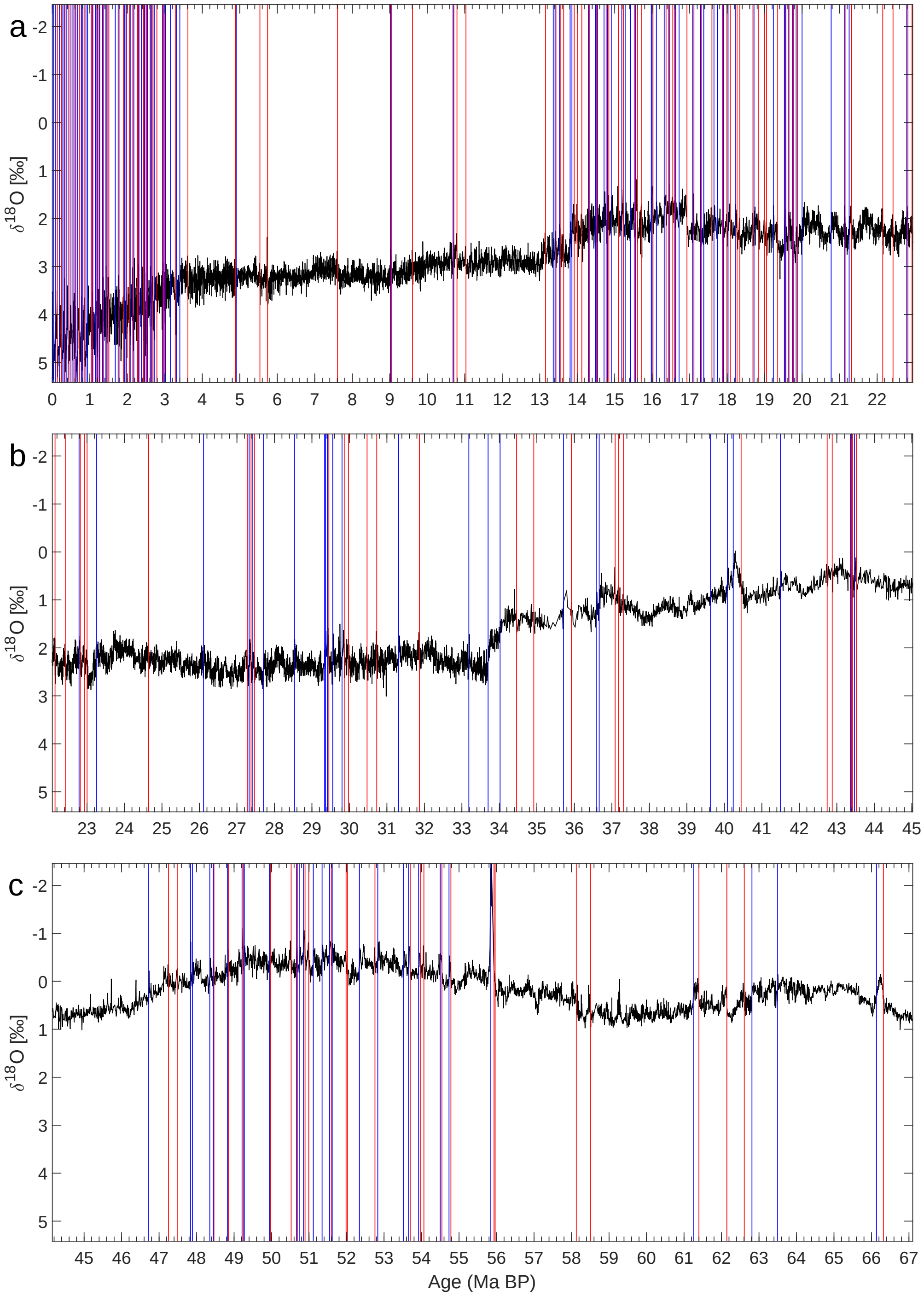

Supplementary Figure S 1. CENOGRID stack of benthic δ^{18}O[62]: (a) 22.9-0 Ma BP; (b) 45-22.1 Ma BP; and (c) 67.1-44.2 Ma BP. Vertical lines represent transitions detected by the KS test[151], with red lines for warming transitions and blue lines for cooling ones. Transitions detected for the entire record using a window length of $0.02 \leq w \leq 2.5$ Myr. The vertical axes are reversed.

U1308 marine sediment core, including detected transitions

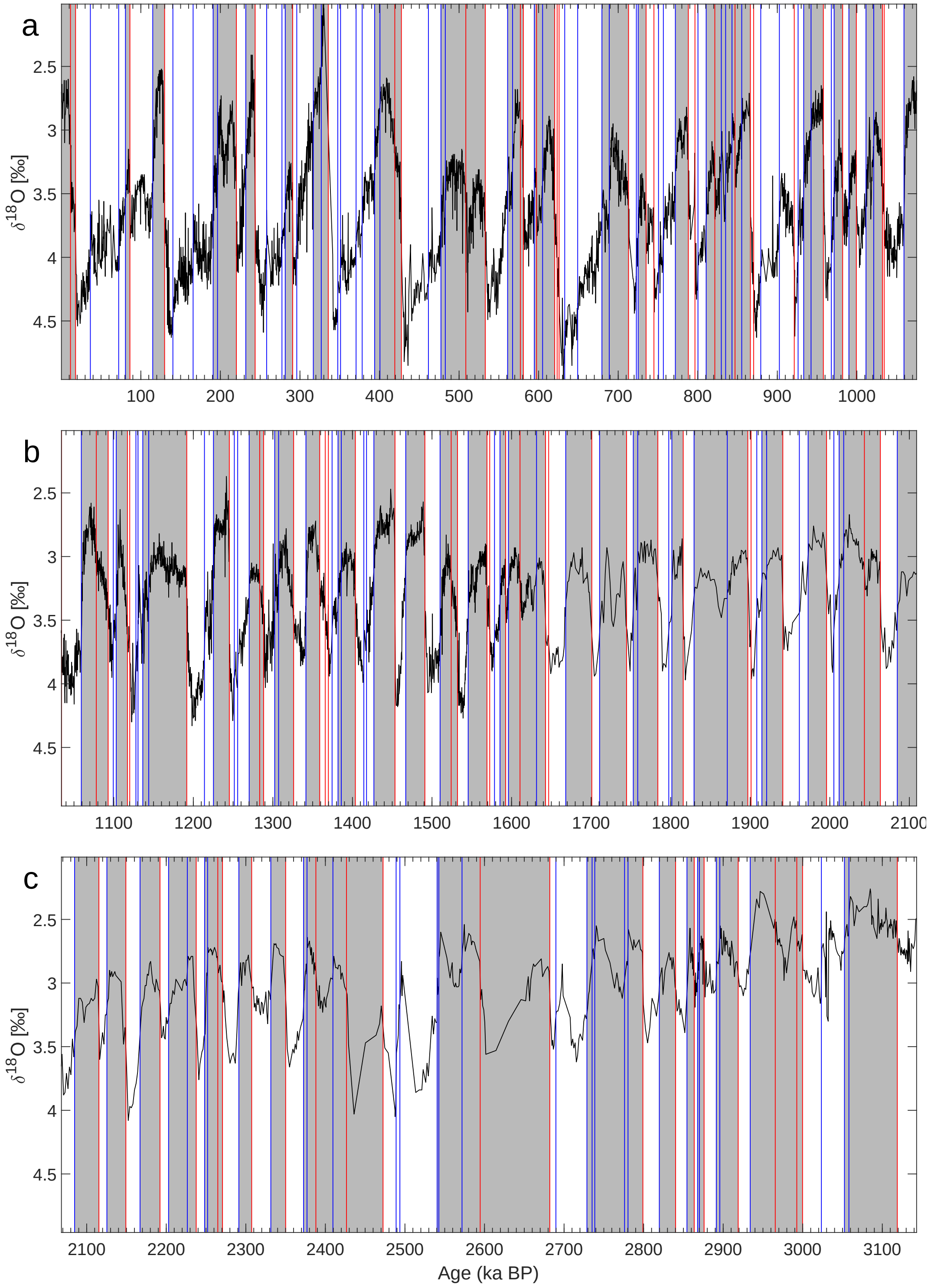

Supplementary Figure S 2. Benthic *Cibicidoides sp.* record in the U1308 marine sediment core δ^{18}O[7]: (a) 1.07-0 Ma BP; (b) 2.11-1.04 Ma BP; and (c) 3.14-2.07 Ma BP. Vertical lines represent transitions detected by the KS test[151], with red lines for warming transitions and blue lines for cooling ones. Vertical axes are reversed. Marine isotope stages (MISs) are shaded, with grey bars representing interglacials (odd-numbered MISs), while white bars represent glacials (even-numbered MISs). See also the analysis of this record by Rousseau et al.[152]

References

1. Dokken, T. M. & Jansen, E. Rapid changes in the mechanism of ocean convection during the last glacial period. *Nature* **401**, 458–461 (1999).
2. Barker, S. *et al.* Early interglacial legacy of deglacial climate instability. *Paleoceanography and Paleoclimatology* **34**, 1455–1475 (2019).
3. Van Kreveld, S. *et al.* Potential links between surging ice sheets, circulation changes, and the Dansgaard-Oeschger cycles in the Irminger Sea, 60–18 kyr. *Paleoceanography* **15**, 425–442 (2000).
4. Waelbroeck, C. *et al.* Consistently dated Atlantic sediment cores over the last 40 thousand years. *Scientific Data* **6**, 1–12 (2019).
5. Dickson, A. J., Austin, W. E., Hall, I. R., Maslin, M. A. & Kucera, M. Centennial-scale evolution of Dansgaard-Oeschger events in the northeast Atlantic Ocean between 39.5 and 56.5 ka BP. *Paleoceanography* **23** (2008).
6. Hodell, D. A., Evans, H. F., Channell, J. E. & Curtis, J. H. Phase relationships of North Atlantic ice-rafted debris and surface-deep climate proxies during the last glacial period. *Quaternary Science Reviews* **29**, 3875–3886 (2010).
7. Hodell, D. A. & Channell, J. E. Mode transitions in Northern Hemisphere glaciation: co-evolution of millennial and orbital variability in Quaternary climate. *Climate of the Past* **12**, 1805–1828 (2016).
8. Hodell, D. A., Channell, J. E., Curtis, J. H., Romero, O. E. & Röhl, U. Onset of "Hudson Strait" Heinrich events in the eastern North Atlantic at the end of the middle Pleistocene transition (~640 ka)? *Paleoceanography* **23** (2008).
9. Harada, N. *et al.* Rapid fluctuation of alkenone temperature in the southwestern Okhotsk Sea during the past 120 ky. *Global and Planetary Change* **53**, 29–46 (2006).
10. Eynaud, F. *et al.* Position of the Polar Front along the western Iberian margin during key cold episodes of the last 45 ka. *Geochemistry, Geophysics, Geosystems* **10** (2009).
11. Davtian, N., Bard, E., Darfeuil, S., Ménot, G. & Rostek, F. The Novel Hydroxylated Tetraether Index RI-OH' as a Sea Surface Temperature Proxy for the 160-45 ka BP Period Off the Iberian Margin. *Paleoceanography and Paleoclimatology* **36**, e2020PA004077 (2021).
12. Naughton, F. *et al.* Wet to dry climatic trend in north-western Iberia within Heinrich events. *Earth and Planetary Science Letters* **284**, 329–342 (2009).
13. Sánchez Goñi, M. F. *et al.* The ACER pollen and charcoal database: a global resource to document vegetation and fire response to abrupt climate changes during the last glacial period. *Earth System Science Data* **9**, 679–695 (2017).
14. Bolton, C. T. *et al.* North Atlantic midlatitude surface-circulation changes through the Plio-Pleistocene intensification of Northern Hemisphere glaciation. *Paleoceanography and Paleoclimatology* **33**, 1186–1205 (2018).
15. Naafs, B. D. A., Voelker, A., Karas, C., Andersen, N. & Sierro, F. Repeated near-collapse of the Pliocene sea surface temperature gradient in the North Atlantic. *Paleoceanography and Paleoclimatology* **35**, e2020PA003905 (2020).
16. Naafs, B., Hefter, J. & Stein, R. Millennial-scale ice rafting events and Hudson Strait Heinrich (-like) Events during the late Pliocene and Pleistocene: a review. *Quaternary Science Reviews* **80**, 1–28 (2013).
17. Voelker, A. H. & Abreu, L. d. A review of abrupt climate change events in the Northeastern Atlantic Ocean (Iberian Margin): latitudinal, longitudinal and vertical gradients. *AGU Geophysical Monograph* (2010).
18. de Abreu, L., Shackleton, N. J., Schönfeld, J., Hall, M. & Chapman, M. Millennial-scale oceanic climate variability off the Western Iberian margin during the last two glacial periods. *Marine Geology* **196**, 1–20 (2003).
19. Salgueiro, E. *et al.* Past circulation along the western Iberian margin: a time slice vision from the Last Glacial to the Holocene. *Quaternary Science Reviews* **106**, 316–329 (2014).
20. Hodell, D. *et al.* Response of Iberian Margin sediments to orbital and suborbital forcing over the past 420 ka. *Paleoceanography* **28**, 185–199 (2013).
21. Martrat, B. *et al.* Four climate cycles of recurring deep and surface water destabilizations on the Iberian margin. *Science* **317**, 502–507 (2007).
22. Shackleton, N. J., Hall, M. A. & Vincent, E. Phase relationships between millennial-scale events 64,000–24,000 years ago. *Paleoceanography* **15**, 565–569 (2000).
23. Hodell, D. *et al.* A reference time scale for Site U1385 (Shackleton Site) on the SW Iberian Margin. *Global and Planetary Change* **133**, 49–64 (2015).

24. Bahr, A. *et al.* Oceanic heat pulses fueling moisture transport towards continental Europe across the mid-Pleistocene transition. *Quaternary Science Reviews* **179**, 48–58 (2018).

25. Voelker, A. H. *et al.* Mediterranean outflow strengthening during northern hemisphere coolings: a salt source for the glacial Atlantic? *Earth and Planetary Science Letters* **245**, 39–55 (2006).

26. Beuscher, S. *et al.* End-member modelling as a tool for climate reconstruction—an Eastern Mediterranean case study. *PLoS One* **12**, e0185136 (2017).

27. Ehrmann, W. & Schmiedl, G. Nature and dynamics of North African humid and dry periods during the last 200,000 years documented in the clay fraction of Eastern Mediterranean deep-sea sediments. *Quaternary Science Reviews* **260**, 106925 (2021).

28. Weldeab, S., Emeis, K.-C., Hemleben, C., Schmiedl, G. & Schulz, H. Spatial productivity variations during formation of sapropels S5 and S6 in the Mediterranean Sea: evidence from Ba contents. *Palaeogeography, Palaeoclimatology, Palaeoecology* **191**, 169–190 (2003).

29. Hendy, I. L., Kennett, J. P., Roark, E. & Ingram, B. L. Apparent synchroneity of submillennial scale climate events between Greenland and Santa Barbara Basin, California from 30–10 ka. *Quaternary Science Reviews* **21**, 1167–1184 (2002).

30. Hendy, I. L. & Kennett, J. P. Latest Quaternary North Pacific surface-water responses imply atmosphere-driven climate instability. *Geology* **27**, 291–294 (1999).

31. Hendy, I. L. & Kennett, J. P. Tropical forcing of North Pacific intermediate water distribution during Late Quaternary rapid climate change? *Quaternary Science Reviews* **22**, 673–689 (2003).

32. Clemens, S. *et al.* Precession-band variance missing from East Asian monsoon runoff. *Nature Communications* **9**, 1–12 (2018).

33. Nürnberg, D., Ziegler, M., Karas, C., Tiedemann, R. & Schmidt, M. W. Interacting Loop Current variability and Mississippi River discharge over the past 400 kyr. *Earth and Planetary Science Letters* **272**, 278–289 (2008).

34. Pichevin, L., Bard, E., Martinez, P. & Billy, I. Evidence of ventilation changes in the Arabian Sea during the late Quaternary: Implication for denitrification and nitrous oxide emission. *Global Biogeochemical Cycles* **21** (2007).

35. Schulz, H., von Rad, U. & Erlenkeuser, H. Correlation between Arabian Sea and Greenland climate oscillations of the past 110,000 years. *Nature* **393**, 54–57 (1998).

36. Deplazes, G. *et al.* Links between tropical rainfall and North Atlantic climate during the last glacial period. *Nature Geoscience* **6**, 213–217 (2013).

37. Deplazes, G. *et al.* Weakening and strengthening of the Indian monsoon during Heinrich events and Dansgaard-Oeschger oscillations. *Paleoceanography* **29**, 99–114 (2014).

38. Zhao, M., Beveridge, N., Shackleton, N., Sarnthein, M. & Eglinton, G. Molecular stratigraphy of cores off northwest Africa: Sea surface temperature history over the last 80 ka. *Paleoceanography* **10**, 661–675 (1995).

39. Lauterbach, S. *et al.* An ~130 kyr record of surface water temperature and δ18O from the northern Bay of Bengal: Investigating the linkage between Heinrich events and Weak Monsoon Intervals in Asia. *Paleoceanography and Paleoclimatology* **35**, e2019PA003646 (2020).

40. Clemens, S. C. *et al.* Remote and local drivers of Pleistocene South Asian summer monsoon precipitation: A test for future predictions. *Science Advances* **7**, eabg3848 (2021).

41. Zarriess, M. & Mackensen, A. The tropical rainbelt and productivity changes off northwest Africa: A 31,000-year high-resolution record. *Marine Micropaleontology* **76**, 76–91 (2010).

42. Zarriess, M. *et al.* Bipolar seesaw in the northeastern tropical atlantic during heinrich stadials. *Geophysical Research Letters* **38** (2011).

43. Jung, S. J., Kroon, D., Ganssen, G., Peeters, F. & Ganeshram, R. Enhanced Arabian Sea intermediate water flow during glacial North Atlantic cold phases. *Earth and Planetary Science Letters* **280**, 220–228 (2009).

44. Rosenthal, Y., Oppo, D. W. & Linsley, B. K. The amplitude and phasing of climate change during the last deglaciation in the Sulu Sea, western equatorial Pacific. *Geophysical Research Letters* **30** (2003).

45. Stott, L., Poulsen, C., Lund, S. & Thunell, R. Super ENSO and global climate oscillations at millennial time scales. *Science* **297**, 222–226 (2002).

46. Saikku, R., Stott, L. & Thunell, R. A bi-polar signal recorded in the western tropical Pacific: Northern and Southern Hemisphere climate records from the Pacific warm pool during the last Ice Age. *Quaternary Science Reviews* **28**, 2374–2385 (2009).

47. Weldeab, S., Lea, D. W., Schneider, R. R. & Andersen, N. 155,000 years of West African monsoon and ocean thermal evolution. *Science* **316**, 1303–1307 (2007).

48. Lea, D. W. *et al.* Paleoclimate history of Galápagos surface waters over the last 135,000 yr. *Quaternary Science Reviews* **25**, 1152–1167 (2006).

49. Mohtadi, M. *et al.* North Atlantic forcing of tropical Indian Ocean climate. *Nature* **509**, 76–80 (2014).

50. Weijers, J. W., Schouten, S., Schefuß, E., Schneider, R. R. & Damste, J. S. S. Disentangling marine, soil and plant organic carbon contributions to continental margin sediments: a multi-proxy approach in a 20,000 year sediment record from the Congo deep-sea fan. *Geochimica et Cosmochimica Acta* **73**, 119–132 (2009).

51. Rampen, S. W. *et al.* Long chain 1, 13-and 1, 15-diols as a potential proxy for palaeotemperature reconstruction. *Geochimica et Cosmochimica Acta* **84**, 204–216 (2012).

52. De Pol-Holz, R. *et al.* Late Quaternary variability of sedimentary nitrogen isotopes in the eastern South Pacific Ocean. *Paleoceanography* **22** (2007).

53. De Deckker, P. *et al.* Climatic evolution in the Australian region over the last 94 ka-spanning human occupancy-, and unveiling the Last Glacial Maximum. *Quaternary Science Reviews* **249**, 106593 (2020).

54. Hodell, D. A., Venz, K. A., Charles, C. D. & Ninnemann, U. S. Pleistocene vertical carbon isotope and carbonate gradients in the South Atlantic sector of the Southern Ocean. *Geochemistry, Geophysics, Geosystems* **4**, 1–19 (2003).

55. Barker, S. & Diz, P. Timing of the descent into the last Ice Age determined by the bipolar seesaw. *Paleoceanography* **29**, 489–507 (2014).

56. Ziegler, M., Diz, P., Hall, I. R. & Zahn, R. Millennial-scale changes in atmospheric CO2 levels linked to the Southern Ocean carbon isotope gradient and dust flux. *Nature Geoscience* **6**, 457–461 (2013).

57. Elderfield, H. *et al.* Evolution of ocean temperature and ice volume through the mid-Pleistocene climate transition. *Science* **337**, 704–709 (2012).

58. Gottschalk, J., Skinner, L. C. & Waelbroeck, C. Contribution of seasonal sub-Antarctic surface water variability to millennial-scale changes in atmospheric CO2 over the last deglaciation and Marine Isotope Stage 3. *Earth and Planetary Science Letters* **411**, 87–99 (2015).

59. Riveiros, N. V. *et al.* Response of South Atlantic deep waters to deglacial warming during Terminations V and I. *Earth and Planetary Science Letters* **298**, 323–333 (2010).

60. Pahnke, K., Zahn, R., Elderfield, H. & Schulz, M. 340,000-year centennial-scale marine record of Southern Hemisphere climatic oscillation. *Science* **301**, 948–952 (2003).

61. Rickaby, R. E. M. & Elderfield, H. Planktonic foraminiferal Cd/Ca: paleonutrients or paleotemperature? *Paleoceanography* **14**, 293–303 (1999).

62. Westerhold, T. *et al.* An astronomically dated record of Earth's climate and its predictability over the last 66 million years. *Science* **369**, 1383–1387 (2020).

63. Rasmussen, S. O. *et al.* A first chronology for the North Greenland Eemian Ice Drilling (NEEM) ice core. *Climate of the Past* **9**, 2713–2730 (2013).

64. Erhardt, T. *et al.* Decadal-scale progression of the onset of Dansgaard–Oeschger warming events. *Climate of the Past* **15**, 811–825 (2019).

65. Gkinis, V. *et al.* A 120,000-year long climate record from a NW-Greenland deep ice core at ultra-high resolution. *Scientific Data* **8**, 1–9 (2021).

66. Rasmussen, S. O. *et al.* A stratigraphic framework for abrupt climatic changes during the Last Glacial period based on three synchronized Greenland ice-core records: refining and extending the INTIMATE event stratigraphy. *Quaternary Science Reviews* **106**, 14–28 (2014).

67. Baumgartner, M. *et al.* NGRIP CH 4 concentration from 120 to 10 kyr before present and its relation to a δ 15 N temperature reconstruction from the same ice core. *Climate of the Past* **10**, 903–920 (2014).

68. Thompson, L. G. *et al.* Tropical climate instability: The last glacial cycle from a Qinghai-Tibetan ice core. *Science* **276**, 1821–1825 (1997).

69. Bazin, L. *et al.* An optimized multi-proxy, multi-site Antarctic ice and gas orbital chronology (AICC2012): 120–800 ka. *Climate of the Past* **9**, 1715–1731 (2013).

70. Vallelonga, P. *et al.* Iron fluxes to Talos Dome, Antarctica, over the past 200 kyr. *Climate of the Past* **9**, 597–604 (2013).

71. Barbante, C. *et al.* One-to-one coupling of glacial climate variability in Greenland and Antarctica. *Nature* **444**, 195–198 (2006).

72. Jouzel, J. *et al.* Orbital and millennial Antarctic climate variability over the past 800,000 years. *Science* **317**, 793–796 (2007).

73. Lüthi, D. *et al.* High-resolution carbon dioxide concentration record 650,000–800,000 years before present. *Nature* **453**, 379–382 (2008).

74. Loulergue, L. *et al.* Orbital and millennial-scale features of atmospheric CH4 over the past 800,000 years. *Nature* **453**, 383–386 (2008).

75. Lambert, F. *et al.* Dust-climate couplings over the past 800,000 years from the EPICA Dome C ice core. *Nature* **452**, 616–619 (2008).

76. Extier, T. *et al.* On the use of δ18Oatm for ice core dating. *Quaternary Science Reviews* **185**, 244–257 (2018).

77. Lambert, F., Bigler, M., Steffensen, J. P., Hutterli, M. & Fischer, H. Centennial mineral dust variability in high-resolution ice core data from Dome C, Antarctica. *Climate of the Past* **8**, 609–623 (2012).

78. Uemura, R. *et al.* Asynchrony between Antarctic temperature and CO2 associated with obliquity over the past 720,000 years. *Nature Communications* **9**, 1–11 (2018).

79. Petit, J.-R. *et al.* Climate and atmospheric history of the past 420,000 years from the Vostok ice core, Antarctica. *Nature* **399**, 429–436 (1999).

80. WAIS Divide Project Members. Precise interpolar phasing of abrupt climate change during the last ice age. *Nature* **520**, 661–665 (2015).

81. Barker, S. *et al.* 800,000 years of abrupt climate variability. *Science* **334**, 347–351 (2011).

82. Moseley, G. E. *et al.* NALPS19: sub-orbital-scale climate variability recorded in northern Alpine speleothems during the last glacial period. *Climate of the Past* **16**, 29–50 (2020).

83. Boch, R. *et al.* NALPS: a precisely dated European climate record 120–60 ka. *Climate of the Past* **7**, 1247–1259 (2011).

84. Genty, D. *et al.* Precise dating of Dansgaard–Oeschger climate oscillations in western Europe from stalagmite data. *Nature* **421**, 833–837 (2003).

85. Genty, D. *et al.* Isotopic characterization of rapid climatic events during OIS3 and OIS4 in Villars Cave stalagmites (SW-France) and correlation with Atlantic and Mediterranean pollen records. *Quaternary Science Reviews* **29**, 2799–2820 (2010).

86. Cheng, H. *et al.* The climatic cyclicity in semiarid-arid central Asia over the past 500,000 years. *Geophysical Research Letters* **39** (2012).

87. Fleitmann, D. *et al.* Timing and climatic impact of Greenland interstadials recorded in stalagmites from northern Turkey. *Geophysical Research Letters* **36** (2009).

88. Cheng, H. *et al.* Climate variations of Central Asia on orbital to millennial timescales. *Scientific Reports* **6**, 1–11 (2016).

89. Lachniet, M. S., Denniston, R. F., Asmerom, Y. & Polyak, V. J. Orbital control of western North America atmospheric circulation and climate over two glacial cycles. *Nature Communications* **5**, 1–8 (2014).

90. Ünal-İmer, E. *et al.* An 80 kyr-long continuous speleothem record from Dim Cave, SW Turkey with paleoclimatic implications for the Eastern Mediterranean. *Scientific Reports* **5**, 1–11 (2015).

91. Moseley, G. E. *et al.* Reconciliation of the Devils Hole climate record with orbital forcing. *Science* **351**, 165–168 (2016).

92. Asmerom, Y., Polyak, V. J. & Burns, S. J. Variable winter moisture in the southwestern United States linked to rapid glacial climate shifts. *Nature Geoscience* **3**, 114–117 (2010).

93. Wang, Y.-J. *et al.* A high-resolution absolute-dated late Pleistocene monsoon record from Hulu Cave, China. *Science* **294**, 2345–2348 (2001).

94. Wagner, J. D. *et al.* Moisture variability in the southwestern United States linked to abrupt glacial climate change. *Nature Geoscience* **3**, 110–113 (2010).

95. Wang, Y. *et al.* Millennial-and orbital-scale changes in the East Asian monsoon over the past 224,000 years. *Nature* **451**, 1090–1093 (2008).

96. Cheng, H. *et al.* The Asian monsoon over the past 640,000 years and ice age terminations. *Nature* **534**, 640–646 (2016).

97. Bar-Matthews, M., Ayalon, A., Gilmour, M., Matthews, A. & Hawkesworth, C. J. Sea–land oxygen isotopic relationships from planktonic foraminifera and speleothems in the Eastern Mediterranean region and their implication for paleorainfall during interglacial intervals. *Geochimica et Cosmochimica Acta* **67**, 3181–3199 (2003).

98. Kathayat, G. *et al.* Indian monsoon variability on millennial-orbital timescales. *Scientific Reports* **6**, 1–7 (2016).

99. Arienzo, M. M. *et al.* Bahamian speleothem reveals temperature decrease associated with Heinrich stadials. *Earth and Planetary Science Letters* **430**, 377–386 (2015).

100. Kelly, M. J. *et al.* High resolution characterization of the Asian Monsoon between 146,000 and 99,000 years BP from Dongge Cave, China and global correlation of events surrounding Termination II. *Palaeogeography, Palaeoclimatology, Palaeoecology* **236**, 20–38 (2006).

101. Burns, S. J., Fleitmann, D., Matter, A., Kramers, J. & Al-Subbary, A. A. Indian Ocean climate and an absolute chronology over Dansgaard/Oeschger events 9 to 13. *Science* **301**, 1365–1367 (2003).

102. Lachniet, M. S. *et al.* Late Quaternary moisture export across Central America and to Greenland: evidence for tropical rainfall variability from Costa Rican stalagmites. *Quaternary Science Reviews* **28**, 3348–3360 (2009).

103. Carolin, S. A. *et al.* Varied response of western Pacific hydrology to climate forcings over the last glacial period. *Science* **340**, 1564–1566 (2013).

104. Mosblech, N. A. *et al.* North Atlantic forcing of Amazonian precipitation during the last ice age. *Nature Geoscience* **5**, 817–820 (2012).

105. Wang, X. *et al.* Hydroclimate changes across the Amazon lowlands over the past 45,000 years. *Nature* **541**, 204–207 (2017).

106. Kanner, L. C., Burns, S. J., Cheng, H. & Edwards, R. L. High-latitude forcing of the South American summer monsoon during the last glacial. *Science* **335**, 570–573 (2012).

107. Stríkis, N. M. *et al.* South American monsoon response to iceberg discharge in the North Atlantic. *Proceedings of the National Academy of Sciences* **115**, 3788–3793 (2018).

108. Denniston, R. F. *et al.* North Atlantic forcing of millennial-scale Indo-Australian monsoon dynamics during the Last Glacial period. *Quaternary Science Reviews* **72**, 159–168 (2013).

109. Cruz, F. W. *et al.* Insolation-driven changes in atmospheric circulation over the past 116,000 years in subtropical Brazil. *Nature* **434**, 63–66 (2005).

110. Whittaker, T. E., Hendy, C. H. & Hellstrom, J. C. Abrupt millennial-scale changes in intensity of Southern Hemisphere westerly winds during marine isotope stages 2–4. *Geology* **39**, 455–458 (2011).

111. Seelos, K., Sirocko, F. & Dietrich, S. A continuous high-resolution dust record for the reconstruction of wind systems in central Europe (Eifel, Western Germany) over the past 133 ka. *Geophysical Research Letters* **36** (2009).

112. Rousseau, D.-D. *et al.* (MIS3 & 2) millennial oscillations in Greenland dust and Eurasian aeolian records–A paleosol perspective. *Quaternary Science Reviews* **169**, 99–113 (2017).

113. Moine, O. *et al.* The impact of Last Glacial climate variability in west-European loess revealed by radiocarbon dating of fossil earthworm granules. *Proceedings of the National Academy of Sciences* **114**, 6209–6214 (2017).

114. Újvári, G. *et al.* AMS 14C and OSL/IRSL dating of the Dunaszekcső loess sequence (Hungary): chronology for 20 to 150 ka and implications for establishing reliable age–depth models for the last 40 ka. *Quaternary Science Reviews* **106**, 140–154 (2014).

115. Sun, Y. *et al.* Influence of Atlantic meridional overturning circulation on the East Asian winter monsoon. *Nature Geoscience* **5**, 46–49 (2012).

116. Sun, Y. *et al.* Astronomical and glacial forcing of East Asian summer monsoon variability. *Quaternary Science Reviews* **115**, 132–142 (2015).

117. Sun, Y. *et al.* High-sedimentation-rate loess records: A new window into understanding orbital-and millennial-scale monsoon variability. *Earth-Science Reviews* **220**, 103731 (2021).

118. Yang, S. & Ding, Z. A 249 kyr stack of eight loess grain size records from northern China documenting millennial-scale climate variability. *Geochemistry, Geophysics, Geosystems* **15**, 798–814 (2014).

119. Sun, Y., Wang, X., Liu, Q. & Clemens, S. C. Impacts of post-depositional processes on rapid monsoon signals recorded by the last glacial loess deposits of northern China. *Earth and Planetary Science Letters* **289**, 171–179 (2010).

120. Hao, Q. *et al.* Delayed build-up of Arctic ice sheets during 400,000-year minima in insolation variability. *Nature* **490**, 393–396 (2012).

121. Sun, Y., Clemens, S. C., An, Z. & Yu, Z. Astronomical timescale and palaeoclimatic implication of stacked 3.6-myr monsoon records from the chinese loess plateau. *Quaternary Science Reviews* **25**, 33–48 (2006).

122. Ding, Z. L. *et al.* Stacked 2.6-Ma grain size record from the Chinese loess based on five sections and correlation with the deep-sea δ18O record. *Paleoceanography* **17**, 5–1 (2002).

123. Melles, M. *et al.* 2.8 million years of Arctic climate change from Lake El'gygytgyn, NE Russia. *Science* **337**, 315–320 (2012).

124. Meyer-Jacob, C. *et al.* Biogeochemical variability during the past 3.6 million years recorded by FTIR spectroscopy in the sediment record of Lake El'gygytgyn, Far East Russian Arctic. *Climate of the Past* **10**, 209–220 (2014).

125. Prokopenko, A. A., Hinnov, L. A., Williams, D. F. & Kuzmin, M. I. Orbital forcing of continental climate during the Pleistocene: a complete astronomically tuned climatic record from Lake Baikal, SE Siberia. *Quaternary Science Reviews* **25**, 3431–3457 (2006).

126. Müller, U. C., Pross, J. & Bibus, E. Vegetation response to rapid climate change in Central Europe during the past 140,000 yr based on evidence from the Füramoos pollen record. *Quaternary Research* **59**, 235–245 (2003).

127. Ampel, L., Wohlfarth, B., Risberg, J. & Veres, D. Paleolimnological response to millennial and centennial scale climate variability during MIS 3 and 2 as suggested by the diatom record in Les Echets, France. *Quaternary Science Reviews* **27**, 1493–1504 (2008).

128. Veres, D. *et al.* Climate-driven changes in lake conditions during late MIS 3 and MIS 2: a high-resolution geochemical record from Les Echets, France. *Boreas* **38**, 230–243 (2009).

129. Reille, M. & De Beaulieu, J. Pollen analysis of a long upper Pleistocene continental sequence in a Velay maar (Massif Central, France). *Palaeogeography, Palaeoclimatology, Palaeoecology* **80**, 35–48 (1990).

130. Benson, L., Lund, S., Negrini, R., Linsley, B. & Zic, M. Response of north American Great basin lakes to Dansgaard–Oeschger oscillations. *Quaternary Science Reviews* **22**, 2239–2251 (2003).

131. Follieri, M., Magri, D. & Sadori, L. Pollen stratigraphical synthesis from Valle di Castiglione (Roma). *Quaternary International* **3**, 81–84 (1989).

132. Tzedakis, P., Hooghiemstra, H. & Pälike, H. The last 1.35 million years at Tenaghi Philippon: revised chronostratigraphy and long-term vegetation trends. *Quaternary Science Reviews* **25**, 3416–3430 (2006).

133. Wagner, B. *et al.* Mediterranean winter rainfall in phase with African monsoons during the past 1.36 million years. *Nature* **573**, 256–260 (2019).

134. Francke, A. *et al.* Sedimentological processes and environmental variability at Lake Ohrid (Macedonia, Albania) between 637 ka and the present. *Biogeosciences* **13**, 1179–1196 (2016).

135. Sadori, L. *et al.* Pollen-based paleoenvironmental and paleoclimatic change at Lake Ohrid (south-eastern Europe) during the past 500 ka. *Biogeosciences* **13**, 1423–1437 (2016).

136. Donders, T. *et al.* 1.36 million years of Mediterranean forest refugium dynamics in response to glacial–interglacial cycle strength. *Proceedings of the National Academy of Sciences* **118** (2021).

137. Allen, J. R. *et al.* Rapid environmental changes in southern Europe during the last glacial period. *Nature* **400**, 740–743 (1999).

138. Allen, J. R., Watts, W. A. & Huntley, B. Weichselian palynostratigraphy, palaeovegetation and palaeoenvironment; the record from Lago Grande di Monticchio, southern Italy. *Quaternary International* **73**, 91–110 (2000).

139. Huntley, B., Watts, W., Allen, J. & Zolitschka, B. Palaeoclimate, chronology and vegetation history of the Weichselian Lateglacial: comparative analysis of data from three cores at Lago Grande di Monticchio, southern Italy. *Quaternary Science Reviews* **18**, 945–960 (1999).

140. Tzedakis, P. *et al.* Ecological thresholds and patterns of millennial-scale climate variability: The response of vegetation in Greece during the last glacial period. *Geology* **32**, 109–112 (2004).

141. Pickarski, N. & Litt, T. A new high-resolution pollen sequence at Lake Van, Turkey: insights into penultimate interglacial–glacial climate change on vegetation history. *Climate of the Past* **13**, 689–710 (2017).

142. Camuera, J. *et al.* Orbital-scale environmental and climatic changes recorded in a new 200,000-year-long multiproxy sedimentary record from Padul, southern Iberian Peninsula. *Quaternary Science Reviews* **198**, 91–114 (2018).

143. Camuera, J. *et al.* Chronological control and centennial-scale climatic subdivisions of the Last Glacial Termination in the western Mediterranean region. *Quaternary Science Reviews* **255**, 106814 (2021).

144. Camuera, J. *et al.* Past 200 kyr hydroclimate variability in the western mediterranean and its connection to the african humid periods. *Scientific Reports* **12**, 1–13 (2022).

145. Miebach, A., Stolzenberger, S., Wacker, L., Hense, A. & Litt, T. A new Dead Sea pollen record reveals the last glacial paleoenvironment of the southern Levant. *Quaternary Science Reviews* **214**, 98–116 (2019).

146. Grimm, E. C. *et al.* Evidence for warm wet Heinrich events in Florida. *Quaternary Science Reviews* **25**, 2197–2211 (2006).

147. Tierney, J. E. *et al.* Northern hemisphere controls on tropical southeast African climate during the past 60,000 years. *Science* **322**, 252–255 (2008).

148. Johnson, T. C. *et al.* A progressively wetter climate in southern East Africa over the past 1.3 million years. *Nature* **537**, 220–224 (2016).

149. Fritz, S. C. *et al.* Quaternary glaciation and hydrologic variation in the South American tropics as reconstructed from the Lake Titicaca drilling project. *Quaternary Research* **68**, 410–420 (2007).

150. Fritz, S. C., Baker, P., Ekdahl, E., Seltzer, G. & Stevens, L. Millennial-scale climate variability during the Last Glacial period in the tropical Andes. *Quaternary Science Reviews* **29**, 1017–1024 (2010).

151. Bagniewski, W., Ghil, M. & Rousseau, D.-D. Automatic detection of abrupt transitions in paleoclimate records. *Chaos* **31**, 113129 (2021).

152. Rousseau, D.-D., Bagniewski, W. & Ghil, M. Abrupt climate changes and the astronomical theory: are they related? *Climate of the Past* **18**, 249–271 (2022).
